# Supplementary material for: Clinician and patient perspectives on the barriers and facilitators to physical rehabilitation in intensive care: a qualitative interview study
Source: BMJ Open. 2023 Nov 8;13(11):e073061. doi: 10.1136/bmjopen-2023-073061 (PMC10632869; doi:10.1136/bmjopen-2023-073061)
Supplement: Supplementary data [file bmjopen-2023-073061supp001.pdf]

Consolidated criteria for reporting qualitative research checklist

Adapted from: TONG, A., SAINSBURY, P. & CRAIG, J. 2007. Consolidated criteria for reporting qualitative research (COREQ): a 32-item checklist for interviews and focus groups. Int J Qual Health Care, 19, 349-57.

Topic	Item no.	Guide questions/ descriptions	Reported on page no.
**Domain 1: Research team and reflexivity**			
Personal Characteristics			
Interviewer/facilitator	1	Which author/s conducted the interview or focus group?	6
Credentials	2	What were the researcher's credentials? E.g. PhD, MD	6
Occupation	3	What was their occupation at the time of the study?	6-7
Gender	4	Was the researcher male or female?	6
Experience and training	5	What experience or training did the researcher have?	6-7
Relationship with participants			
Relationship established	6	Was a relationship established prior to study commencement?	6-7
Participant knowledge of the interviewer	7	What did the participants know about the researcher? e.g. personal goals, reasons for doing the research	6-7
Interviewer characteristics	8	What characteristics were reported about the interviewer/facilitator? e.g. Bias, assumptions, reasons and interests in the research topic	6-7
**Domain 2: study design**			
Theoretical framework			
Methodological orientation and Theory	9	What methodological orientation was stated to underpin the study? e.g. grounded theory, discourse analysis, ethnography, phenomenology, content analysis	4
Participant selection			
Sampling	10	How were participants selected? e.g. purposive, convenience, consecutive, snowball	5
Method of approach	11	How were participants approached? e.g. face-to-face, telephone, mail, email	5
Sample size	12	How many participants were in the study?	8
Non-participation	13	How many people refused to participate or dropped out? Reasons?	8
Setting			
Setting of data collection	14	Where was the data collected? e.g. home, clinic, workplace	6
Presence of non-participants	15	Was anyone else present besides the participants and researchers?	6
Description of sample	16	What are the important characteristics of the sample? e.g. demographic data, date	9-10
Data collection			

Interview guide	17	Were questions, prompts, guides provided by the authors? Was it pilot tested?	6
Repeat interviews	18	Were repeat interviews carried out? If yes, how many?	6
Audio/visual recording	19	Did the research use audio or visual recording to collect the data?	6
Field notes	20	Were field notes made during and/or after the interview or focus group?	7
Duration	21	What was the duration of the interviews or focus group?	8
Data saturation	22	Was data saturation discussed?	5-6
Transcripts returned	23	Were transcripts returned to participants for comment and/or correction?	6
**Domain 3: analysis and findings**			
Data analysis			
Number of data coders	24	How many data coders coded the data?	7
Description of the coding tree	25	Did authors provide a description of the coding tree?	Supplementary material
Derivation of themes	26	Were themes identified in advance or derived from the data?	7
Software	27	What software, if applicable, was used to manage the data?	7
Participant checking	28	Did participants provide feedback on the findings?	5-6
Reporting			
Quotations presented	29	Were participant quotations presented to illustrate the themes / findings? Was each quotation identified? e.g. participant number	11-17, supplementary material
Data and findings consistent	30	Was there consistency between the data presented and the findings?	10-18, supplementary material
Clarity of major themes	31	Were major themes clearly presented in the findings?	10-18, supplementary material
Clarity of minor themes	32	Is there a description of diverse cases or discussion of minor themes?	10-18
