# Supplementary material for: Clinician and patient perspectives on the barriers and facilitators to physical rehabilitation in intensive care: a qualitative interview study
Source: BMJ Open. 2023 Nov 8;13(11):e073061. doi: 10.1136/bmjopen-2023-073061 (PMC10632869; doi:10.1136/bmjopen-2023-073061)
Supplement: Supplementary data [file bmjopen-2023-073061supp002.pdf]

Supplemental file 2

1. Patient / relative / carer topic guide.

2. Initial thematic framework

3. Table with summary of themes, sub-categories and verbatim quotes with participant numbers

Interview topic guide: Patient representative

Introduction:

- We value your opinions/what you think about these questions; there's not necessarily a right or wrong answer; it's not a test.

Examples of general probes that may be used

- Tell me more about that.
- Why do you think that?
- Have you got any examples?

If unable to answer open question:

- Some people have said this … [e.g. a known barrier from the literature if not already mentioned – see below for specific examples] …what do you think?

If participant unable to define early mobilisation:

- We are defining early mobilisation as something the patient does with 'their own muscle strength and control' including activities such as:
    - Moving in bed
    - Exercises
    - Sitting on the edge of the bed,
    - Standing
    - Marching on the spot,
    - Transferring from bed to chair
    - Walking…

…all whilst patients are on intensive care.

Woodbridge HR, *et al. BMJ Open* 2023; 13:e073061. doi: 10.1136/bmjopen-2023-073061

Interview questions

General questions:

1. Please tell me about your experience on the ICU.

2. Please tell me about physiotherapy and mobilisation (moving around and getting up and out of bed) on the ICU.
    a. What you would think of as early mobilisation?
    b. What early mobilisation did you do? How often did you do it?
    c. How was it decided that it was the right time for you to start mobilising on the ICU?

3. Do you think that early mobilisation was carried out enough?

Focused questions:

4. When you were asked if you wanted to mobilise, did you have any concerns over risks or problems that might occur?
    Potential question-specific prompts: not being well enough; fall; lines and drains/breathing tube falling out.

5. Was there anything that stopped or delayed you from mobilising on the ICU?

    Potential question-specific prompts: Feeling too unwell; lines and tubes; not enough staff.

6. What kind of things stopped mobilisation from happening more often on the ICU you were on?

Potential question-specific prompts: Not a priority; team did not work together; team did not have enough teaching.

7. Why do you think the staff on you ICU wanted you to mobilise and what do you think the benefits were?

   Example of potential question-specific prompts: Physical benefits; leave ICU sooner, prevent long-term complications.

8. In your experience, what things helped you to mobilise on the ICU?

   Potential question-specific prompts: Different professions working together; feeling well.

9. What kind of things do you think could be improved or changed to help mobilisation to happen more often on your ICU and to overcome some of the problems you mentioned earlier?

   Potential question-specific prompts: Better team communication; staff getting more teaching.

10. This question will explore other areas that previous interviews have brought up as important: e.g. other people have mentioned this… what do you think?

11. Is there anything else you'd like to say about what stops early mobilisation on the ICU and what could make it happen more often?

Initial thematic framework.

BARRIERS

1. Perceived risk of mobilising certain patients [SAFETY/RISK]

1.1 Airway and attachments

1.2 Patient instability
1.3 Patient type
1.4 Patient cognitive state
1.5 Patient medical status

1.6 Clinicians' perception of readiness to mobilise
1.7 Other

2 Patient's or their family member's reluctance for mobilisation

2.1 Clinician opinion of patient's or their family member's reluctance for mobilisation
2.2 Patients not feeling ready or motivated for mobilisation
2.3 Poor communication from clinicians

2.4 Aspects of the ICU environment not promoting mobilisation to patients

2.5 Other

3 Team working and unit culture/staff experience/ resources

3.1 Culture/Lesser priority
3.2 Roles and responsibilities
3.3 Lack of leadership
3.4 Staff experience
3.5 Lack of knowledge
3.6 Lack of resources
3.7 Logistics/ Other interventions
3.8 Nurse environment e.g. HDU/toilet
3.9 Other

FACILITATORS

4 Practical changes to how mobilisation was carried out.

4.1 Patient and family engagement
4.2 Mob treatment specific/functional rehabilitation
4.3 Use of protocols to facilitate clinical implementation of mobilisation
4.4 Patient opinion on how clinicians should communicate with them.
4.5 Specific patient motivators

4.6 Equipment/environment

4.7 Other changes/optimal practice

5. Improvements in team working and culture/clinician specific

5.1 Leadership
5.2 Team Communication
5.3 Experienced staff
5.4 Improved staffing/resources
5.5 Other team working
5.6 Prioritise
5.7 Education
5.8 Other culture change

6. Patient characteristics that made it easier to mobilise

6.1 Pre-morbid/general characteristics status
6.2 Acute/admission-related status
6.3 Other

7. Risks, benefits and other

7.1 Risks

7.2 Benefits

7.3 Other/irrelevant

Table: *Summary of themes and sub-categories*

Themes	Sub-categories	Quote number	Participant quote
1. Safety and physiological concerns	1.1 Airway, lines and attachments	1	*"I can't think who, they said an intensive care patient looks like little spiders in a web, and I agree with it. Like literally they have got tubes and attachments out everywhere." (Therapist 1)*
	1.2 Particular patient groups	2	*"…the types of patients we have have multiple and complex injuries, they're not straightforward patients to mobilise anyway…" (Therapist 3)*
	1.3 Physiological instability or dependence on organ support	3<br>4<br>5	*"…it's mainly blood pressure related for me, or their resp[iratory] rate. If I don't think they're going to tolerate mobilising, and if it's going to cause more harm than good." (Therapist 2)*<br>*"So anybody who's on an inotrope vasopressor is, as far as I'm concerned, not safe to be mobilised… they're more likely to have a postural hypertension that would result in injury to them." (Doctor 2)*<br>*"…whilst in itself [vasoactive drugs are] often not a reason to prevent ongoing rehab especially in junior staff it's a significant source of anxiety of doubling or trebling the dose of a medicine to keep your blood pressure up, without some form of kind of very clear guidance and encouragement that this is okay and it will return to normal following [rehabilitation] treatment." (Nurse 5)*
	1.4 Patient's ability to actively participate	6<br>7<br>8	*"And after that incident I think that was the first time I actually cried, because it hit me that "Yes, the nurses are right, I am not able to just get up and move like I would if I had been healthy," you know, so that was very traumatic for me…" (Patient 11).*<br>*"I think I would have felt very vulnerable anyhow, [be]cause suddenly you are just weak as a baby." (Patient 7)*<br>*"Their cognitive state is a massive thing as well. How alert are they if they've only just been woken up from sedation or if they've had a neurological event or, you know, whatever reason, that could affect their cognition." (Therapist 6)*
	1.5 Clinician perception of readiness to begin rehabilitation	9<br>10	*"…the perception, [clinicians] might think that, because this patient is dependent on a particular type of organ support, this patient is not suitable for mobilisation. So these boundaries and barriers needs to be broken." (Doctor 3)*<br>*"I think it's probably the fear of the unwell patient, you know, we, they're in ITU therefore they must be the most unwell people in the hospital. And I think it's that kind of mentality and the fact we attach them to fifteen hundred things…" (Therapist 7)*

		11<br><br><br>12<br><br><br>13<br><br><br><br>14	*"…happy to cause no harm, or kind of, and no perceived harm by not mobilising someone but actively getting up and causing harm is a, always going to be a significant anxiety for staff…" (Nurse 5)*<br>*"We love keeping the numbers normal, we love the sense of security that we maintain as normal physiology as we can, so that is why, a junior nurse would be more worried if she gets any change in the patient's state after mobilisation…" (Doctor 1)*<br>*"…intensive care doctors and nurses may also be quite, well, I shouldn't say "quite" but there is a part in us that is controlling the situation and so, you know, trying to mobilise the patient may also be a bit of a paradigm shift in our own mind of, you know, this complete control over the situation and over this patient." (Doctor 4)*<br>*"…so we kind of reset our expectations about normality and that is doing some sort of exercise when they ambulate because it is – it is to a critically ill patient it is an exercise, that we may see some events happen and as long as it is the range of acceptance, we can just modify our targets, and continue to mobilise." (Doctor 1)*
2. Patient participation and engagement	2.1 Patient reluctance to participate in rehabilitation	15<br><br>16	*"...well I'm in ICU... you're having intensive care, don't rock the boat by making things worse by trying to get out of bed." (Patient 8)*<br>*"…there were times when I simply didn't want to do it… Depression, … lack of energy, lack of spirits really …" (Patient 7)*
	2.2 Communication between patients and clinicians	17<br><br>18	*"I would say to any nurse or any staff working in ICU… keep up that reassurance with patients because it's quite a scary experience…" (Patient 8)*<br>*"[The consultant] pushed me beyond what I mentally thought was physically possible. I didn't believe that I could do that and of course, perhaps it's the nature of my personality, but I responded to that. Others may not have responded to that, I can't say." (Patient 3)*
	2.3 Patient engagement in planning rehabilitation	19	*"So, alongside that, we've also made like goal setting sheets that can go up by the patient's bed, so then when they sit up, when they sit upright in bed, they can see them. I draw a smiley face when they've completed one…" (Therapist 2)*
	2.4 Including activities meaningful to patients	20	*"Looking at therapy in a slightly different way and finding an activity that's meaningful to [patients], whether that's personal care or leisure activities, and through that encouraging them to… engage in that activity and then helping them to see the therapeutic value of that." (Therapist 4)*
	2.5 Identify key patient motivators	21	*"…if they can see what's in it for them, that they're gaining in dignity and all of that, they might cooperate more." (Relative 2)*

	2.6 The role of family	22	*"I remember the first time I took a few steps, the nurse said to me, "Well we'll do it with your husband," so my husband stood on one side and said, "We'll go for a walk with your husband," ... So it was most amazing feeling ever, you know? So everything, kind of in my head everything shut down; the nurse went away, the ward went away, it was just me and my husband going for a walk." (Patient 11)*
3. Clinician experience and knowledge	3.1 Amount of experience and support	23	*"I've had instances where it's mostly been junior people and it's terrifying. But for someone then to have a senior position helping you, that's so much better." (Nurse 2)*
	3.2 Lack of training, knowledge and skills	24	*"It doesn't happen because… we are not aware enough yet how important it is, or how much difference it could make, so it's not embedded in our thinking and in our behaviour well…" (Doctor 4)*
	3.3 Interdisciplinary team education and training	25	*"The education as well is important because you need to get people to understand what they're doing and to value it, so that they do it with passion and with skill." (Doctor 1)*
4. Teamwork	4.1 Team culture and attitudes	26	*"But a lot of it's just to do with the attitude of the individual staff member, how proactive they are and how much they believe in mobilisation as a kind of key thing" (Nurse 5)*
	4.2 Perception of roles and responsibilities	27<br><br><br><br><br>28<br><br>29<br><br>30	*"…I think the consultant's role is very important and it doesn't just include saying, "Mobilise the patient". It includes making sure that mobilisation happens and making sure that the team are, like every single member of the team is comfortable and understands the decision, and the risks related to it and understands that I am there to back them up if something happens." (Doctor 1)*<br>*"I've found that it's taken a long time for me to be accepted and for them to actually accept my opinion might be right…" (Therapist 2)*<br>*"I always felt like it was, it was very much seen it was the physio job to do anything related to moving the patient so even getting them out of bed." (Therapist 7)*<br>*"...good teamwork is really helpful, and actually a really good symbiotic relationship between the nursing staff and the therapy staff is really key." (Therapist 4)*
	4.3 Definition and delivery of rehabilitation	31<br>32<br><br><br><br>33	*"…mobilisation for me in ITU is hoisting somebody into a chair." (Nurse 4)*<br>*"...rehabilitation is not, you know, 20 minutes with the physio or the OT every day. Really good rehabilitation is a 24 hour approach, and that – part of that is positioning a patient in bed. Part of that is ensuring the patient gets the right nutrition as well as looking at the actual physical things that they're doing." (Therapist 4)*<br>*"...a different mentality within intensive care and to think, well actually, you know, we need to begin the rehab process all together from day one, and if a patient can be encouraged to do something they should be given the time and the opportunity to do that." (Therapist 4)*

	4.4 Staffing and logistics	34	*"…you start breaks at 9.30, 10.00. You finish the breaks about 12.00,1.00, so then it's not until the afternoon that people are free to help." (Nurse 2)*
5. Equipment and environment	5.1 Rehabilitation equipment	35	*"…equipment wise. You know, it's the age old problem isn't it, more of it, better ways to fix it, more money, so we've got the equipment, you know, got backup hoists." (Therapist 7)*
	5.2 ICU environment	36<br><br><br>37	*"there's just something about the environment which makes you think that you need to stay in the bed and that you shouldn't be moving around… whereas on a general ward, you don't want to be in the bed, you want to get out." (Patient 8)*<br>*"…you can see some bright lights and monitors, you can hear monitors going off, but you don't have the, "Crash, bang, wallops!" that you get in a general ward... but it's a capsule and a bubble, it's a weird feeling... "People think it's like being in a spaceship" and I thought, "That's such a good description" and that's how it did feel." (Patient 8)*
6. Risks and benefits of rehabilitation on intensive care	6.1 Clinician perception of risks	38<br><br>39	*"Falls, removal of lines and tubes and then causing bleeding, vasovagal episodes, it's actually a risky thing to mobilise an ITU patient, anything can go wrong…" (Nurse 3)*<br>*"I like mobilising patients. The more attachments the better… Because I like the challenge!" (Therapist 2)*
	6.2 Patient perception of risks	40	*"I never felt scared, I felt that the physiotherapist that was orchestrating the movement was sort of holding on to me to begin with and I never felt I was going to fall down…" (Patient 1)*
	6.3 Physical benefits	41	*"It might help their movement and I feel the more they mobilise the more their muscles are good. The more you make them sit out of the bed and stand they can stand on their feet better." (Nurse 1)*
	6.4 Psychological benefits	42	*"…the important thing is you sense that you're not just lying there waiting to die. ...so you are… you are… coming back to being a human being that wants to live." (Patient 7)*
