# Supplementary material for: Clinician and patient perspectives on the barriers and facilitators to physical rehabilitation in intensive care: a qualitative interview study
Source: BMJ Open. 2023 Nov 8;13(11):e073061. doi: 10.1136/bmjopen-2023-073061 (PMC10632869; doi:10.1136/bmjopen-2023-073061)
Supplement: Author's manuscript [file bmjopen-2023-073061.draft_revisions.pdf]

BMJ Open

BMJ Open is committed to open peer review. As part of this commitment we make the peer review history of every article we publish publicly available.

When an article is published we post the peer reviewers' comments and the authors' responses online. We also post the versions of the paper that were used during peer review. These are the versions that the peer review comments apply to.

The versions of the paper that follow are the versions that were submitted during the peer review process. They are not the versions of record or the final published versions. They should not be cited or distributed as the published version of this manuscript.

BMJ Open is an open access journal and the full, final, typeset and author-corrected version of record of the manuscript is available on our site with no access controls, subscription charges or pay-per-view fees (http://bmjopen.bmj.com).

If you have any questions on BMJ Open's open peer review process please email info.bmjopen@bmj.com

BMJ Open

Clinician and patient perspectives on the barriers and facilitators to physical rehabilitation in intensive care: a qualitative interview study

Journal:	*BMJ Open*
Manuscript ID	bmjopen-2023-073061
Article Type:	Original research
Date Submitted by the Author:	08-Mar-2023
Complete List of Authors:	Woodbridge, Huw; Imperial College Healthcare NHS Trust; Imperial College London, Department of Surgery and Cancer<br>Norton, Christine; Kings College London, Florence Nightingale Faculty of Nursing, Midwifery and Palliative Care<br>Jones, Mandy ; Brunel University London<br>Brett, Stephen; Imperial College Healthcare NHS Trust, Anaesthetics and Intensive Care; Imperial College London, Department of Surgery and Cancer<br>Alexander, Caroline; Imperial College Healthcare NHS Trust, Therapies; Imperial College London, MSk Labs<br>Gordon, Anthony; Imperial College London, Department of Surgery and Cancer; Imperial College Healthcare NHS Trust
Keywords:	Adult intensive & critical care < INTENSIVE & CRITICAL CARE, QUALITATIVE RESEARCH, REHABILITATION MEDICINE

SCHOLARONE™
Manuscripts

I, the Submitting Author has the right to grant and does grant on behalf of all authors of the Work (as defined in the below author licence), an exclusive licence and/or a non-exclusive licence for contributions from authors who are: i) UK Crown employees; ii) where BMJ has agreed a CC-BY licence shall apply, and/or iii) in accordance with the terms applicable for US Federal Government officers or employees acting as part of their official duties; on a worldwide, perpetual, irrevocable, royalty-free basis to BMJ Publishing Group Ltd ("BMJ") its licensees and where the relevant Journal is co-owned by BMJ to the co-owners of the Journal, to publish the Work in this journal and any other BMJ products and to exploit all rights, as set out in our licence.

The Submitting Author accepts and understands that any supply made under these terms is made by BMJ to the Submitting Author unless you are acting as an employee on behalf of your employer or a postgraduate student of an affiliated institution which is paying any applicable article publishing charge ("APC") for Open Access articles. Where the Submitting Author wishes to make the Work available on an Open Access basis (and intends to pay the relevant APC), the terms of reuse of such Open Access shall be governed by a Creative Commons licence – details of these licences and which Creative Commons licence will apply to this Work are set out in our licence referred to above.

Other than as permitted in any relevant BMJ Author's Self Archiving Policies, I confirm this Work has not been accepted for publication elsewhere, is not being considered for publication elsewhere and does not duplicate material already published. I confirm all authors consent to publication of this Work and authorise the granting of this licence.

For peer review only

Clinician and patient perspectives on the barriers and facilitators to physical rehabilitation in intensive care: a qualitative interview study.

Authors

Huw R. Woodbridge*

- Imperial College Healthcare NHS Trust, London, UK; Imperial College London, London, UK.

Christine Norton

- King's College London, London, UK.

Mandy Jones

- Brunel University London, London, UK.

Stephen J. Brett

- Imperial College Healthcare NHS Trust, London, UK; Imperial College London, London, UK.

Caroline M. Alexander

- Imperial College Healthcare NHS Trust, London, UK; Imperial College London, London, UK.

Anthony C. Gordon

- Imperial College London, London, UK; Imperial College Healthcare NHS Trust, London, UK.

*Corresponding author: Physiotherapy Department, St Mary's Hospital, Praed Street, London W2 1NY. Email: huw.woodbridge@nhs.net Telephone: 0203 312 1794.

For peer review only

Keywords: Adult intensive & critical care; qualitative research; rehabilitation medicine.

Word count: 5381

Abstract

Objectives

To explore patient, relative/carer and clinician perceptions of barriers to early physical rehabilitation in intensive care units (ICU) within an associated group of hospitals in the United Kingdom (UK) and how they can be overcome.

Design

Qualitative study using semi-structured interviews and thematic framework analysis.

Setting

Four ICUs over three hospital sites in London, UK.

Participants

Former ICU patients or their relatives/carers with personal experience of ICU rehabilitation. ICU clinicians, including doctors, nurses, physiotherapists and occupational therapists, involved in the delivery of physical rehabilitation or decisions over its initiation.

Interventions

Nil

Primary and secondary outcomes measures

Views and experiences on the barriers and facilitators to ICU physical rehabilitation.

Results

Interviews were carried out with 11 former patients, 3 family members and 16 clinicians. The themes generated related to: safety and physiological concerns; patient participation and engagement; clinician experience and knowledge; teamwork; equipment and environment; and risks and benefits of rehabilitation in intensive care. The overarching theme related to how barriers can be overcome by moving away from a multidisciplinary approach and towards an interdisciplinary, patient-centred approach to ICU physical rehabilitation. This involves a change in working model from ICU clinicians having separate

For peer review only

responsibilities to one where all parties have a shared aim of providing physical rehabilitation.

Conclusions

The results have revealed barriers that can be modified to improve rehabilitation delivery in an ICU. Interdisciplinary working could overcome many of these barriers to optimise recovery from critical illness.

For peer review only

Strengths and limitations of this study

- This study explored the perspectives of multiple stakeholders including intensive care clinicians from different professions, with a range of experience. Importantly, the views of former ICU patients and their family members were sought to obtain a full range of perspectives on the barriers to ICU rehabilitation.
- Thematic framework analysis was used which enables a systematic approach to organising data, facilitating in-depth exploration of the range of views within themes and between participant groups.
- Patient and family recall of their experiences may have been impacted by the time from intensive care admission to interview, however interviews took place at the first follow up opportunity to minimise this effect.
- Efforts were made to gain a range of perspectives using purposive sampling; however, fewer family members or carers took part in this study than former ICU patients.

Introduction

The importance of physical rehabilitation of critically ill patients has been recognised because of the prevalence of acute muscle weakness and wasting [1-3], and longer-term substantial physical disability measured in this patient group [2 4 5]. Physical rehabilitation consists of physical activity interventions (typically mobilising in or away from the bed) that are begun once a patient has reached physiological stability [6-8]. Beginning physical

rehabilitation at an appropriate dose whilst patients are still in an intensive care unit (ICU) can improve physical function whilst in hospital and expedite discharge [9], although implementing rehabilitation at a higher dose is not necessarily beneficial [10]. However, when measured, there is concern that the actual amount of formal physical rehabilitation delivered and patient participation in exercise whilst in intensive care are low [11-13].

Studies have previously measured the barriers to implementing rehabilitation, the majority of which use a quantitative approach [14]. However, a qualitative approach is better-suited to exploring interpersonal relations [15] and therefore potential barriers relating to team working and patient interactions. Where a qualitative approach has been used, issues of communication and differences in opinion between clinicians [16-21] and difficulty in providing rehabilitation in an environment where demands on staff and patient time change quickly have been highlighted [19 22 23]. However, the lack of rehabilitation in intensive care continues despite this current understanding of the barriers. Importantly, there is a lack of in-depth knowledge of barriers in a United Kingdom (UK) setting, which includes views of multiple stakeholders such as ICU clinicians from different professions involved in implementing rehabilitation, as well as patients and family members [18 24-26].

The objective of this study therefore, was to explore service user (patients or their relatives/carers) and clinician perceptions of barriers to early physical rehabilitation in ICUs within an associated group of hospitals in the UK and how they can be overcome.

Methods

A qualitative study using semi-structured interviews was conducted based on the approach recommended by the National Centre for Social Research. This is based upon critical realism and interpretivism using the framework approach to analysis [27]. Ethical approval was gained from the London – Bloomsbury Research Ethics Committee (17/LO/0362) and written, informed consent was gained from all participants. This study is reported in line with the consolidated criteria for reporting qualitative research [28] (supplemental file 1).

The study was based at a UK National Health Service (NHS) hospital trust in London, which has four ICUs for adult patients across three hospital sites, each of which has different

For peer review only

referring specialities. Purposive sampling [29 30] was used to recruit a range of service users (former ICU patients and their family members/carers) and the hospital's ICU clinicians from the different ICU settings, from different professional groups with a range of experience levels. Clinicians were included if they were doctors (senior trainees [registrars/fellows] or consultants), nurses, occupational therapists or physiotherapists with at least two months of ICU experience and who had experience of rehabilitation treatments or deciding when they should be initiated. Clinicians were approached via adverts in meetings, posted in staff areas or via general group or more targeted emails. Former ICU patients and family members were included if they had personal experience of physical rehabilitation whist in ICU. Participants were excluded if they could not attend an in-person interview, if they felt unable to participate in English, if they were less than 18 years of age or unable to give informed consent. Patients or their relatives/carers were approached via local ICU patient support groups and follow up clinics. Estimates were that 30 participants would be required to gain a sufficient range of perspectives, with sampling ending once apparent data saturation had been reached. Data saturation was defined a priori as when no new themes of barriers and facilitators were evident from interviews, as decided by the study steering group. During data collection, when the interviewer felt no new themes were being discussed, the latest version of the initial thematic framework was shown to the final clinician and patient participant as a sense check to see whether they could identify any additional themes that had been missed. Following this, the initial thematic framework was reviewed by clinical colleagues from all four professional groups included in the study, as well as steering group members who were former ICU patients, to discuss if any obvious themes were missing.

For peer review only

Interviews took place at the hospital site and only included the interviewer and the individual participant. Each participant took part in one semi-structured interview. Before the interview began, participants were asked for demographic data then the interview proceeded using a topic guide (supplemental file 2), designed by the research steering group which included the input of former ICU patients. The interview was piloted with both clinicians and former patients. The format of the interview was a conversation, where wording was not fixed and prompts were used to gain greater depth of understanding of participant views and experiences. Participants were asked to define physical rehabilitation

themselves, however, the study was designed based on rehabilitation consisting of mobilisation treatments ranging from exercises and movement in the bed, to mobilising out of bed and walking [8]. Participants were informed of this if they had difficulty defining rehabilitation or if their definition was markedly different from how the study conceptualised rehabilitation. Each interview was recorded and then transcribed verbatim. Transcripts were not returned to participants for review in line with current thinking about usefulness of this approach [31].

All interviews were carried out by one interviewer (HRW) who is a male physiotherapist, working full-time on the research study as part of work towards a doctorate, with training in qualitative research methods. The interviewer had previous clinical experience at several of the ICUs that were settings for this study, including working alongside some of the clinician participants, but not the patients or their relatives/carers. The researcher therefore had previous experiences of barriers and facilitators of rehabilitation in the study setting. These influences were taken into account using a reflexive diary before and after data collection, which was then considered during the analysis process.

Thematic framework analysis [32 33] was used to produce themes based on the interview transcript data. This involves drawing up an initial list of themes that summarised all the interview data (supplemental file 2). Data were then arranged in a framework table which structured what each participant had said about each initial theme in an easily accessible form. This facilitated the production of a final set of themes and subthemes and comparison of how these vary between groups of participants. Analysis was facilitated by the use of NVivo 11 software (QSR International) and carried out by the first author (HRW). A second researcher (MJ) reviewed 10% of interview transcripts and confirmed that they matched the initial set of themes. At several stages during the analysis process, the research steering group met to review the data, discuss uncertainties over formation of themes and as a check on the process. Descriptive statistics were used to summarise demographic data using IBM SPSS Statistics. Continuous data were tested for normality using the Shapiro-Wilk test and non-normally distributed data described using median and interquartile range, and normally distributed data described using mean and standard deviation.

For peer review only

Patient and public involvement

Former ICU patients were members of the research steering group. These patient representatives edited the wording of recruitment materials and inputted into the design of the topic guide. They also assisted the interviewer (HRW) to practice interview technique and were involved in reviewing the initial thematic framework, as part of data saturation checks.

Results

Recruited participants included 16 clinicians, from a range of professions, with a range of experience in different settings (Table 1). Eleven former ICU patients and three family members/caregivers participated, all with experience of ICU rehabilitation (Table 2). Initially, 53 potential participants expressed interest in taking part, of whom 30 were recruited before data saturation was achieved. Five declined or were not available for interview, three did not respond further after initial contact, data saturation was achieved before four were recruited and 11 were not recruited as others were chosen instead to gain a greater range of views, as per the purposive sampling strategy. Interviews lasted for a mean 43 minutes (standard deviation ± 11 minutes).

For peer review only

Table 1: Clinician participant demographics	
	**Clinicians (N=16)**
Age, mean (± SD)	34 (8.6)
Gender: female, n (%)	12 (75)
Profession, n (%)	
Doctor	4 (25)
Nurse	5 (31)
Therapist (physiotherapist or occupational therapist)	7 (44)
Seniority, n (%)	
Team leader	9 (56)
Senior clinician	6 (38)
Junior clinician	1 (6)
Number of years of ICU experience, median (IQR)	6 (1-15)
Number of years of clinical healthcare experience, mean (± SD)	11 (8)
Place of work*, n (%)	
Intensive care 1	5 (31)
Intensive care 2	6 (38)
Intensive care 3	5 (31)
Intensive care 4	5 (31)
Involvement in physical rehabilitation, n (%)	
Participating in the decision over whether a patient is stable enough to mobilise.	16 (100)
Leading rehabilitation treatment	10 (63)
Assisting with rehabilitation treatment	12 (75)

SD= standard deviation; IQR=interquartile range. *Some clinicians work on more than one intensive care unit.

For peer review only

Table 2: Patient and caregiver participant demographics	
	**Former ICU patients and caregivers (N=14)**
Service user participants:	
Patients, n (%)	11 (79)
Caregivers, n (%)	3 (21)
Age, mean (±SD)	65 (10.7)
Male, n (%)	10 (71)
Patient ICU length of stay in days* (n=11), mean (±SD)	15 (10.8)
Patient stated reason for admission (n=11), n (%)	
Aortic dissection	1 (9)
Cardiac arrest	1 (9)
Gastrointestinal	1 (9)
Organ failure	1 (9)
Septic shock	1 (9)
Surgery	5 (45)
Trauma	1 (9)
Site where ICU was experienced, n (%)	
Intensive care 1	2 (14)
Intensive care 2	11 (79)
Intensive care 3	0 (0)
Intensive care 4	1 (7)
Highest level of physical rehabilitation experienced, n (%)	
Moving in bed	2 (14)
Sitting in a chair	6 (43)
Walking	6 (43)
SD= standard deviation. *Two participants reported their length of stay as approximate.	

The study themes and subthemes are described in detail below. The supplementary material illustrates these themes and subthemes with verbatim quotes from participants, with participant numbers (see supplemental file 2 to link quote numbers with data).

1. *Safety and physiological concerns*

Participant concerns over the safety of rehabilitation were reported as a barrier to rehabilitation. This included the risk of dislodging lines and attachments (such as ventilator tubing and femoral lines (quote 1). However, some participants did not perceive this as a barrier, if careful planning and also organisation of the bed space environment was carried out. For example, avoiding the use of femoral vascular catheters as access for haemofiltration or planning breaks in haemofiltration could enable rehabilitation. Endotracheal tubes or airways that had been difficult to insert, were also cited as barriers,

For peer review only

with the difficulty of titrating sedation for a balance between tube tolerance and patient alertness cited as one explanation.

Clinician participants identified particular patient groups with barriers to rehabilitation because they felt they were at an increased risk or they presented additional logistical challenges, such as those with multiple traumatic injuries (quote 2). They suggested that patients admitted after surgery could have certain surgical precautions which presented logistical issues contacting different teams to gain clarity over safety of rehabilitation. Despite this, patients in ICU after elective surgery could have received pre-operative education or pre-planned rehabilitation programmes, both facilitating rehabilitation post-operatively.

Physiological instability, such as instances of respiratory distress or cardiovascular instability were reported as preventing rehabilitation treatments.

> *"…it's mainly blood pressure related for me, or their resp[iratory] rate. If I don't think they're going to tolerate mobilising, and if it's going to cause more harm than good." (Therapist 2, quote 3)*

Dependence on organ support, such as the amount of respiratory support or vasoactive drugs were also identified as barriers. Clinician opinion ranged from perceiving patients receiving vasoactive drugs as a contraindication to rehabilitation (quote 4), to others who considered rehabilitation possible if a low or weaning dose was used or if the patient was less severely unwell, for example if vasoactive drugs were being used for epidural-induced hypotension. Risk relating to hypotension during rehabilitation was suggested to relate to anxiety from junior staff about managing vasoactive drugs during mobilisation (quote 5). Participants suggested potential organ support barriers should always be discussed with the ICU doctors and also advocated actively sedating patients less.

Patient participants sometimes reported feeling too unwell to actively participate in rehabilitation. Some patients reported profound feelings of weakness, making their bodies feel 'like a lead weight', which came as a surprise when they first tried to get up and was linked with feelings of vulnerability (quotes 6 and 7). These participants did then identify a

For peer review only

time in their recovery where these symptoms subsided to the point where they could then participate.

Additionally, level of alertness, confusion and agitation, cognitive impairments and personality disorders were all cited as barriers by clinicians (quote 8). Some patients and relatives recalled experiences of delirium and hallucinations as profound influences on their recovery in general.

The difference between clinicians' perception of safety and a patient's readiness to begin rehabilitation was expressed as a barrier (quote 9). Some explanations included clinician fear of the unwell patient and the risk of perceived harm which caused anxiety for some (quote 10).

> *"…happy to cause no harm, or kind of, and no perceived harm by not mobilising someone but actively getting up and causing harm is a, always going to be a significant anxiety for staff…" (Nurse 5, quote 11)*

This was linked to clinician need for control over the physiological numbers, potentially leading to a reluctance to reduce that control by moving a patient out of bed (quote 12). One doctor suggested that a paradigm shift was required to address this barrier (quote 13). Another doctor said they modified targets for acceptable changes in physiological observations (such as blood pressure), to reassure other clinicians that mobility was still safe (quote 14).

2. *Patient participation and engagement*

Clinician participants reported that patients may be reluctant to participate in rehabilitation. Patient participant responses ranged from reporting enthusiastic engagement in rehabilitation, to not wishing to mobilise out of bed. Reasons cited for their reluctance included not wanting to do something perceived as potentially worsening their condition (quote 15). Furthermore, a lack of incentive or motivation to engage was discussed, as well as a feeling of weakness, which some found difficult to accept.

For peer review only

"...there were times when I simply didn't want to do it... Depression, ... lack of energy, lack of spirits really ..." (Patient 7, quote 16)

Suboptimal communication between patients and clinicians was felt to be a barrier to rehabilitation. Suggested reasons included the little time spent by clinicians discussing rehabilitation, difficulty communicating rehabilitation goals and some sometimes showed a lack of empathy. Suggested ways of overcoming these issues included maximising a patient's ability to communicate, giving more reassurance, building up trust, showing kindness and helping patients to feel safe (quote 17). Patients valued humour from staff and felt rapport was aided by staff continuity. Patients and relatives recommended that when a patient was reluctant to mobilise, an encouraging and diplomatic approach balanced with assertiveness from clinicians to "push" patients (quote 18).

Participants recommended that strategies to improve patient engagement in rehabilitation should always be patient-specific. Other suggestions included promoting sleep at night, involving patients in planning a rehabilitation timetable, goal setting and using outcome measures to demonstrate progress (quote 19). Furthermore, education for patients and relatives at the appropriate time, around the importance of rehabilitation was suggested.

Further facilitators included the use of meaningful activities and identifying key patient motivators. The importance of tailoring rehabilitation to include activities meaningful to patients (such as functional tasks and personal care activities based on previous interests) were identified to facilitate engagement within a context more readily understood by patients.

"Looking at therapy in a slightly different way and finding an activity that's meaningful to [patients], whether that's personal care or leisure activities, and through that encouraging them to... engage in that activity and then helping them to see the therapeutic value of that." (Therapist 4, quote 20)

Recognising key patient motivators such as gaining independence and dignity by being able to do more for themselves was also suggested (quote 21). Patients reported being motivated through their improvement during rehabilitation sessions, almost as a proxy for

For peer review only

improvement from critical illness. Patient qualities of resilience, determination and a positive mental attitude were reported as a facilitator.

The role of family was discussed as both a barrier and facilitator. Some instances were reported where relatives could be reluctant for patient participation in rehabilitation. When this was discussed with patient and relative participants, responses ranged from an understanding of why this happens, to a strong disbelief that this could be the case. The role of family in encouraging patients was discussed, with some highlighting how they were motivated to improve mobility to help their family member feel better (quote 22).

3. *Clinician experience and knowledge*

Clinician participants discussed the experience and knowledge of those carrying out rehabilitation. A lack of experience, confidence and senior support were cited as barriers (quote 23). However, some therapists also proposed those clinicians with more experience could pose a barrier. They suggested some more experienced nurses may perceive rehabilitation as outside of their role or may have spent more time in an environment where rehabilitation was not a priority. Opinions over experience as a facilitator also varied. Some emphasised that a team with the right skill mix (including adequate senior support) was important, with a nurse suggesting having more confident staff freed up time for rehabilitation. However, some therapists reported that more inexperienced nurses could be a facilitator as they have received recent training in rehabilitation. One therapist cited enthusiasm as being more important than experience to facilitate rehabilitation.

A lack of training and knowledge, including about the importance of rehabilitation, organisation and planning of sessions and therapeutic manual handling were suggested as important factors.

> *"It doesn't happen because… we are not aware enough yet how important it is, or how much difference it could make, so it's not embedded in our thinking and in our behaviour…" (Doctor 4, quote 24)*

A popular strategy to address these barriers was through education and training for the ICU interdisciplinary team, such as through study days and experiential learning (quote 25).

For peer review only

Additionally, the use of a rehabilitation policy and guidelines to drive implementation and aid less experienced clinicians know when to begin rehabilitation was discussed.

4. Teamwork

Discussion of teamwork covered team culture, clinician roles, rehabilitation definitions and logistics. A lack of a rehabilitation culture leading to some staff having a less proactive attitude to rehabilitation delivery was discussed.

> *"But a lot of it's just to do with the attitude of the individual staff member, how proactive they are and how much they believe in mobilisation as a kind of key thing" (Nurse 5, quote 26)*

One explanatory factor was a lack of medical leadership. Participants suggested promoting a culture where an interdisciplinary team works together to promote rehabilitation as routine and important, would facilitate implementation. A less hierarchical culture would encourage proactive team planning and problem solving, with medical leadership again emphasised as key (quote 27).

Another key barrier to rehabilitation was differences in opinion between professions over roles and responsibilities (quote 28). Some reported that rehabilitation was perceived as only a therapist's job (quote 29). Therapists reported that there could be a lack of understanding of their role or their other responsibilities, for example, covering other clinical areas in addition to the ICU. To overcome this, clinicians suggested promoting teamwork where separate responsibilities were acknowledged and there was a willingness to crossover professional roles, with therapists empowering nurses to facilitate rehabilitation (quote 30).

Differences in opinions over roles and responsibilities were impacted upon by variation in how rehabilitation was defined and delivered. This in itself may explain some of the difficulty in promoting a proactive rehabilitation culture. Participants noted that doctors and nurses sometimes limited their definition of rehabilitation to a patient sitting out in a chair (quote 31). Conversely, occupational therapist (OT) participants widened the concept of

For peer review only

rehabilitation to encompass a twenty-four hour interdisciplinary approach utilising functional tasks.

> *"...rehabilitation is not, you know, 20 minutes with the physio or the OT every day. Really good rehabilitation is a 24 hour approach, and that – part of that is positioning a patient in bed. Part of that is ensuring the patient gets the right nutrition as well as looking at the actual physical things that they're doing." (Therapist 4, quote 32)*

This may increase patient engagement and interdisciplinary involvement, by helping staff to incorporate more rehabilitation activities during the course of their normal duties, for example during personal care activities (quote 33).

Finally, lack of staff and logistical difficulties in implementing rehabilitation were suggested as barriers. Greater investment in staffing and utilisation of healthcare support workers was suggested to address this. Logistical concerns covered the number of staff required and the duration of a rehabilitation session in competition with other unit procedures. Logistical barriers also concerned a difficulty in timing around nurses' rest breaks and staffing ratios (quote 34). Within the study ICUs, once a patient's illness severity decreased to a certain level, the nursing staffing ratio fell from one nurse to one patient to one nurse to two patients, coinciding with a potential increase in readiness for rehabilitation. Potential strategies to address these concerns include proactive planning of sessions, for example during morning team briefings. Additionally, a change to working patterns to build in more time for rehabilitation to occur was suggested.

5. *Equipment and environment*

A lack of working specialist rehabilitation equipment was highlighted as a barrier (quote 35). Clinicians advocated greater investment and suggested the whole team to take ownership of ensuring equipment was fixed or to find funding sources for equipment replacement. Environmental concerns firstly covered practical limitations such as space to move rehabilitation equipment around the bedspace. Furthermore, a patient highlighted the nature of the ICU environment itself did not encourage them to move out of bed (quote 36).

For peer review only

"…you can see some bright lights and monitors, you can hear monitors going off, but you don't have the, "Crash, bang, wallops!" that you get in a general ward... but it's a capsule and a bubble, it's a weird feeling... "People think it's like being in a spaceship" and I thought, "That's such a good description" and that's how it did feel." (Patient 8, quote 37)

6. Risks and benefits of rehabilitation in intensive care

Opinions over risks and benefits were explored, which closely related to safety, knowledge and attitude towards rehabilitation. Clinician ideas about risks resembled the safety issues from theme one, however, this did not necessarily mean a reluctance to mobilise (quotes 38 and 39). Most patients and relatives reported they had not worried about the risks of mobilising whilst in an ICU (quote 40), although some had experienced things such as dizziness and one reported passing out. Considering benefits, reported physical benefits of rehabilitation focused on the acute impact of improving physical function, including in preparation for recovery on the wards (quote 41). Suggested psychological benefits for the patient included helping mood and wellbeing and restoring a sense of dignity.

"…the important thing is you sense that you're not just lying there waiting to die. ...so you are… you are… coming back to being a human being that wants to live." (Patient 7, quote 42)

Finally, several clinicians reported how a benefit of patient rehabilitation was the encouragement and sense of achievement it provided for staff.

The overarching theme for how to overcome barriers to physical ICU rehabilitation related to moving from a multidisciplinary approach where different professions work together but have separate responsibilities; towards a patient-centred, interdisciplinary team approach. This was where all parties have a shared aim of providing physical rehabilitation (quotes 27 and 30). This can facilitate clinicians working together to develop a shared understanding of the definition of rehabilitation, so patients can participate in activities that are more meaningful. Furthermore, an agreement can be developed among the team, about the benefits and risks, the optimum way to deliver rehabilitation and when it is safe to start.

For peer review only

This can then help different professions to collaborate to help to overcome barriers related to team working and to improve the ICU environment.

Discussion

This study has provided an in-depth exploration of the views of multi-professional ICU clinicians and was strengthened by including former ICU patients and their relatives, adding to the knowledge of overcoming barriers to ICU physical rehabilitation. Primarily this is suggested to be through a change in approach to team working, from a multidisciplinary to an interdisciplinary and patient-centred approach. This means moving from a multidisciplinary way of working where a team is made up of different professions working on their distinct priorities [34-36], to an interdisciplinary approach where a team of different professions work together with ICU rehabilitation a priority for all. This therefore emphasises a shift from rehabilitation primarily being the focus of therapy staff, to one where all team members have joint accountability and identify this as a key aspect to their work, contributing in overlapping ways but also in ways relevant to their professional skills and knowledge [37 38]. This change in perspective could facilitate a change in opinion over the definition and delivery of rehabilitation towards an interdisciplinary, 24-hour approach that includes activities meaningful to patients to facilitate engagement. An interdisciplinary working model has previously been used to facilitate more efficient and effective care during critical illness and in general rehabilitation delivery. Reported outcomes have included more coordinated inter-professional working and enhanced delivery of appropriate patient care [35 39 40].

It is interesting to see that several of the themes of barriers and facilitators to ICU rehabilitation are similar to previous qualitative studies. This includes themes of safety [19 20 23 24 41], patient engagement [25], knowledge and experience [17-22] and team work [16 18-21 23-25 41]. Patient reports of experiencing feelings of weakness and vulnerability in this present study have also previously been identified [26 42] where their vulnerability may be explained, at least in part, by patients adjusting to being critically ill whilst having little or no memory of their deterioration into critical illness [43]. In our study, clinicians expressed differences in opinion over roles and responsibilities towards rehabilitation as well as safety concerns for initiating

treatment. Staff confidence in rehabilitation provision may contribute towards differences in viewpoints and engagement. This may be partially explained through differences in personality traits between those more or less able to make pragmatic concessions to adjust to the limitations of the working environment to ensure reasonable care is delivered [44]. This would therefore represent an important factor to address with staff when overcoming barriers to rehabilitation.

This study has added to previous knowledge in several ways. Interestingly and perhaps surprisingly, less clinical experience was highlighted as a potential facilitator of rehabilitation. Some therapists reported that more inexperienced nurses have received recent training in rehabilitation and one therapist cited enthusiasm as more important than experience. Additionally, perceptions of the content of rehabilitation were notable. Some viewed rehabilitation as being limited to sitting in a chair. This can contribute to a limited scope of rehabilitation practice [22] and may have contributed to the lack of rehabilitation culture reported by some participants in this study. The occupational therapists emphasised the inclusion of personal care activities as part of ICU rehabilitation delivered at any point in the day by any profession, to facilitate a more positive rehabilitation culture. This is supported by Laerkner, et al. [45] who compared the views of nurses and patients in Denmark, and found nurses recommended incorporating familiar activities into rehabilitation and patients emphasised the importance of empathy and compromise from clinicians. Whilst patients in this present study agreed with Laerkner's recommendations of clinician-patient communication, they also emphasised that at times, a more assertive approach from clinicians in encouraging rehabilitation is desirable.

The findings from this study focus us to create a patient-centred interdisciplinary approach to rehabilitation. This involves considering how clinicians communicate with patients and broadening the definition of rehabilitation to include functional tasks that are meaningful to patients. Furthermore, broadening delivery of rehabilitation to a 24-hour holistic approach that includes family members, with a focus on prioritising patient-reported motivators of independence and dignity and to progress back towards normality. Facilitating this change in a multifaceted ICU environment would benefit from using implementation and improvement science methodology, where co-design by ICU clinicians from different professions, as well as service users can be employed. This change in practise should be

For peer review only

evaluated not only in terms of whether it improves rehabilitation delivery without impacting patient safety, but also in terms of how these changes influence other ICU procedures and working practices [46].

Limitations of this study include the potential for poor recall from patient or relative/carer participants as the time from ICU admission to interview was not recorded. However, as participants were usually recruited at their first ICU follow up appointment, this was unlikely to be an extended time. Furthermore, language limitations of some participants sometimes made it difficult to discern the exact point they were making during analysis, therefore although this demonstrates diversity within the sample, some finer detail may have been lost. The method of approach may have meant that more patients actively engaged in the issues being evaluated were recruited. Those patients not attending follow up appointments may have had different opinions. Finally, pragmatic constrictions meant few family members were recruited and more patients who had experienced one of the ICU sites were involved.

In conclusion, this exploration of a range of clinician and patient perspective suggested a patient-centred, interdisciplinary approach to implementing ICU physical rehabilitation. These findings constitute a starting point for optimising rehabilitation delivery through improvement and implementation science.

Acknowledgements

We thank staff at the research site for their support in facilitating recruitment and data collection. Data from this study has previously been presented at the Intensive Care Society State of the Art meeting 2018[47] and 2021[48], the 6th European Conference on Weaning and Rehabilitation in Critically Ill Patients 2018 (abstract available on page 61 of an online conference book, https://kuleuvencongres.be/icurecovery18/documents/conference-book-icurecovery.pdf), Physiotherapy UK conference 2018[49] and the North West London Research Symposium for Health Professions 2018.

Funding statement

This report is independent research arising from a Clinical Doctoral Research Fellowship, awarded to HRW, (ICA-CDRF-2015-01-026), supported by the National Institute for Health

For peer review only

Research (NIHR) and Health Education England. The views expressed in this publication are those of the authors and not necessarily those of the NHS, the NIHR, Health Education England or the Department of Health. We acknowledge the support of the NIHR Clinical Research Network and infrastructure support for this research was provided by the NIHR Imperial Biomedical Research Centre (BRC). HRW is supported by the NIHR Imperial BRC.

Competing interests statement

HRW reports personal lecture fees from Brunel University and the University of East London. CN reports personal lecture fees from Janssen Pharmaceuticals and Web MD. CMA is a National Institute of Health Research PhD fellowship committee member, although not at the time that funding was awarded for this study. ACG reports salary support from an NIHR Research Professorship (RP-2015-06-018) and research support from the NIHR Imperial BRC.

Author contributions

All authors made substantial contributions to the conception and design of this work. HRW carried out recruitment of participants, data collection and had main responsibility for carrying out analysis. MJ, CN and CMA assisted in checking development of themes and all authors advised on interpretation of data. HRW drafted this report and all authors revised it critically for important content and approved the final published version. All authors are accountable for the accuracy and integrity of the work.

References

1. Puthucheary ZA, Rawal J, McPhail M, et al. Acute skeletal muscle wasting in critical illness. *JAMA* 2013;310:1591-600.
2. Fan E, Dowdy DW, Colantuoni E, et al. Physical complications in acute lung injury survivors: a two-year longitudinal prospective study. *Crit Care Med* 2014;42:849-59.
3. Latronico N, Herridge M, Hopkins RO, et al. The ICM research agenda on intensive care unit-acquired weakness. *Intensive Care Med* 2017;43:1270-81.
4. Herridge MS, Tansey CM, Matte A, et al. Functional disability 5 years after acute respiratory distress syndrome. *N Engl J Med* 2011;364:1293-304.
5. Hodgson CL, Udy AA, Bailey M, et al. The impact of disability in survivors of critical illness. *Intensive Care Med* 2017;43:992-1001.
6. Bailey P, Thomsen GE, Spuhler VJ, et al. Early activity is feasible and safe in respiratory failure patients. *Crit Care Med* 2007;35:139-45.
7. Clarissa C, Salisbury L, Rodgers S, et al. Early mobilisation in mechanically ventilated patients: a systematic integrative review of definitions and activities. *J Intensive Care* 2019;7:3.

For peer review only

8. Hodgson CL, Stiller K, Needham DM, et al. Expert consensus and recommendations on safety criteria for active mobilization of mechanically ventilated critically ill adults. *Crit Care* 2014;18:658.
9. Wang YT, Lang JK, Haines KJ, et al. Physical Rehabilitation in the ICU: A Systematic Review and Meta-Analysis. *Crit Care Med* 2021. https://www.ncbi.nlm.nih.gov/pubmed/34406169 (accessed 8th January 2022).
10. Team Study Investigators, Anzics Clinical Trials Group, Hodgson CL, et al. Early Active Mobilization during Mechanical Ventilation in the ICU. *N Engl J Med* 2022;387:1747-58.
11. Connolly BA, Mortimore JL, Douiri A, et al. Low Levels of Physical Activity During Critical Illness and Weaning: The Evidence-Reality Gap. *J Intensive Care Med* 2019;34:818-27.
12. Nydahl P, Ruhl AP, Bartoszek G, et al. Early mobilization of mechanically ventilated patients: a 1-day point-prevalence study in Germany. *Crit Care Med* 2014;42:1178-86.
13. Bakhru RN, Wiebe DJ, McWilliams DJ, et al. An Environmental Scan for Early Mobilization Practices in U.S. ICUs. *Crit Care Med* 2015;43:2360-9.
14. Parry SM, Knight LD, Connolly B, et al. Factors influencing physical activity and rehabilitation in survivors of critical illness: a systematic review of quantitative and qualitative studies. *Intensive Care Med* 2017;43:531-42.
15. Ormston R, Spencer L, Barnard M, et al. The foundations of qualitative research. In: Ritchie J, Lewis J, McNaughton Nicholls C, et al., eds. Qualitative research practice A guide for social science students and researchers. 2nd ed. London: SAGE 2014:1-25.
16. Barber EA, Everard T, Holland AE, et al. Barriers and facilitators to early mobilisation in Intensive Care: a qualitative study. *Aust Crit Care* 2015;28:177-82; quiz 83.
17. Anekwe DE, Milner SC, Bussieres A, et al. Intensive care unit clinicians identify many barriers to, and facilitators of, early mobilisation: a qualitative study using the Theoretical Domains Framework. *J Physiother* 2020;66:120-27.
18. Chaplin T, McLuskey J. What influences nurses' decision to mobilise the critically ill patient? *Nurs Crit Care* 2020;25:353-59.
19. Parry SM, Remedios L, Denehy L, et al. What factors affect implementation of early rehabilitation into intensive care unit practice? A qualitative study with clinicians. *J Crit Care* 2017;38:137-43.
20. Holdsworth C, Haines KJ, Francis JJ, et al. Mobilization of ventilated patients in the intensive care unit: An elicitation study using the theory of planned behavior. *J Crit Care* 2015;30:1243-50.
21. Goddard SL, Lorencatto F, Koo E, et al. Barriers and facilitators to early rehabilitation in mechanically ventilated patients-a theory-driven interview study. *J Intensive Care* 2018;6:4.
22. Krupp AE, Ehlenbach WJ, King B. Factors Nurses in the Intensive Care Unit Consider When Making Decisions About Patient Mobility. *Am J Crit Care* 2019;28:281-89.
23. Eakin MN, Ugbah L, Arnautovic T, et al. Implementing and sustaining an early rehabilitation program in a medical intensive care unit: A qualitative analysis. *J Crit Care* 2015;30:698-704.
24. Curtis L, Irwin J. Ambulation of patients who are mechanically ventilated: nurses' views. *Nurs Manag* 2017;24:34-39.
25. Williams N, Flynn M. An exploratory study of physiotherapists' views of early rehabilitation in critically ill patients. *Physiother Pract Res* 2013;34:93-102.
26. van Willigen Z, Ostler C, Thackray D, et al. Patient and family experience of physical rehabilitation on the intensive care unit: a qualitative exploration. *Physiotherapy* 2020;109:102-10.
27. Ritchie J, Lewis J, McNaughton Nicholls C, et al. Qualitative research practice. A guide for social science students and researchers. 2nd ed. London: SAGE 2014.
28. Tong A, Sainsbury P, Craig J. Consolidated criteria for reporting qualitative research (COREQ): a 32-item checklist for interviews and focus groups. *Int J Qual Health Care* 2007;19:349-57.
29. Bryman A. Social research methods. 5th ed. Oxford: Oxford University Press 2016.

For peer review only

30. Ritchie J, Lewis J, Elam G, et al. Designing and selecting samples. In: Ritchie J, Lewis J, McNaughton Nicholls C, et al., eds. Qualitative research practice A guide for social science students and researchers. 2nd ed. London: SAGE 2014:111-45.
31. McConnell-Henry T, Chapman Y, Francis K. Member checking and Heideggerian phenomenology: a redundant component. *Nurse Res* 2011;18:28-37.
32. Spencer L, Ritchie J, Ormston R, et al. Analysis: Principles and processes. In: Ritchie J, Lewis J, McNaughton Nicholls C, et al., eds. Qualitative research practice A guide for social science students and researchers. 2nd ed. London: SAGE 2014:269-93.
33. Spencer L, Ritchie J, O'Connor W, et al. Analysis in practice. In: Ritchie J, Lewis J, McNaughton Nicholls C, et al., eds. Qualitative research practice A guide for social science students and researchers. 2nd ed. London: SAGE 2014:295-345.
34. Choi BC, Pak AW. Multidisciplinarity, interdisciplinarity and transdisciplinarity in health research, services, education and policy: 1. Definitions, objectives, and evidence of effectiveness. *Clin Invest Med* 2006;29:351-64.
35. Korner M. Interprofessional teamwork in medical rehabilitation: a comparison of multidisciplinary and interdisciplinary team approach. *Clin Rehabil* 2010;24:745-55.
36. Jennings C, Astin F. A multidisciplinary approach to prevention. *Eur J Prev Cardiol* 2017;24:77-87.
37. Bronstein LR. A model for interdisciplinary collaboration. *Soc Work* 2003;48:297-306.
38. Petri L. Concept analysis of interdisciplinary collaboration. *Nurs Forum* 2010;45:73-82.
39. Irwin RS, Flaherty HM, French CT, et al. Interdisciplinary collaboration: the slogan that must be achieved for models of delivering critical care to be successful. *Chest* 2012;142:1611-19.
40. Urisman T, Garcia A, Harris HW. Impact of surgical intensive care unit interdisciplinary rounds on interprofessional collaboration and quality of care: Mixed qualitative-quantitative study. *Intensive Crit Care Nurs* 2018;44:18-23.
41. Liew SM, Mordiffi SZ, Ong YJA, et al. Nurses' perceptions of early mobilisation in the adult Intensive Care Unit: A qualitative study. *Intensive Crit Care Nurs* 2021;66:103039.
42. Soderberg A, Karlsson V, Ahlberg BM, et al. From fear to fight: Patients experiences of early mobilization in intensive care. A qualitative interview study. *Physiother Theory Pract* 2020. https://www.tandfonline.com/doi/full/10.1080/09593985.2020.1799460 (accessed 12 July 2021).
43. Corner EJ, Murray EJ, Brett SJ. Qualitative, grounded theory exploration of patients' experience of early mobilisation, rehabilitation and recovery after critical illness. *BMJ Open* 2019;9:e026348.
44. D'Lima DM, Murray EJ, Brett SJ. Perceptions of Risk and Safety in the ICU: A Qualitative Study of Cognitive Processes Relating to Staffing. *Crit Care Med* 2018;46:60-70.
45. Laerkner E, Egerod I, Olesen F, et al. Negotiated mobilisation: An ethnographic exploration of nurse-patient interactions in an intensive care unit. *J Clin Nurs* 2019;28:2329-39.
46. Skivington K, Matthews L, Simpson SA, et al. A new framework for developing and evaluating complex interventions: update of Medical Research Council guidance. *BMJ* 2021;374:n2061.
47. Woodbridge HR, Norton C, Jones M, et al. EP.076, Clinician and patient perceptions of the barriers and facilitators of mobilisation of intensive care patients in a UK NHS Trust. Conference abstract. *J Intensive Care Soc* 2019;20:59.
48. Woodbridge HR, Alexander CM, Jones M, et al. Exploring the barriers to early physical rehabilitation and investigating its safety in critically ill patients receiving vasoactive drugs. Rising Star - ICS Gold Medal. Conference abstract. *Journal of the Intensive Care Society* 2022;23:199.
49. Woodbridge HR, Norton C, Alexander CM, et al. Exploring clinician's perceptions about the barriers and facilitators of early mobilisation of patients on intensive care. Conference abstract. *Physiotherapy* 2019;105:e119-e20.

For peer review only

Consolidated criteria for reporting qualitative research checklist

Adapted from: TONG, A., SAINSBURY, P. & CRAIG, J. 2007. Consolidated criteria for reporting qualitative research (COREQ): a 32-item checklist for interviews and focus groups. Int J Qual Health Care, 19, 349-57.

**Topic**	**Item no.**	**Guide questions/ descriptions**	**Reported on page no.**
**Domain 1: Research team and reflexivity**			
Personal Characteristics			
Interviewer/facilitator	1	Which author/s conducted the interview or focus group?	6
Credentials	2	What were the researcher's credentials? E.g. PhD, MD	6
Occupation	3	What was their occupation at the time of the study?	6
Gender	4	Was the researcher male or female?	6
Experience and training	5	What experience or training did the researcher have?	6
Relationship with participants			
Relationship established	6	Was a relationship established prior to study commencement?	6
Participant knowledge of the interviewer	7	What did the participants know about the researcher? e.g. personal goals, reasons for doing the research	6
Interviewer characteristics	8	What characteristics were reported about the interviewer/facilitator? e.g. Bias, assumptions, reasons and interests in the research topic	6
**Domain 2: study design**			
Theoretical framework			
Methodological orientation and Theory	9	What methodological orientation was stated to underpin the study? e.g. grounded theory, discourse analysis, ethnography, phenomenology, content analysis	4
Participant selection			
Sampling	10	How were participants selected? e.g. purposive, convenience, consecutive, snowball	5
Method of approach	11	How were participants approached? e.g. face-to-face, telephone, mail, email	5
Sample size	12	How many participants were in the study?	7
Non-participation	13	How many people refused to participate or dropped out? Reasons?	7
Setting			
Setting of data collection	14	Where was the data collected? e.g. home, clinic, workplace	5
Presence of non-participants	15	Was anyone else present besides the participants and researchers?	5
Description of sample	16	What are the important characteristics of the sample? e.g. demographic data, date	8-9
Data collection			

For peer review only

Interview guide	17	Were questions, prompts, guides provided by the authors? Was it pilot tested?	5
Repeat interviews	18	Were repeat interviews carried out? If yes, how many?	5
Audio/visual recording	19	Did the research use audio or visual recording to collect the data?	6
Field notes	20	Were field notes made during and/or after the interview or focus group?	6
Duration	21	What was the duration of the interviews or focus group?	7
Data saturation	22	Was data saturation discussed?	5
Transcripts returned	23	Were transcripts returned to participants for comment and/or correction?	6
**Domain 3: analysis and findings**			
Data analysis			
Number of data coders	24	How many data coders coded the data?	6
Description of the coding tree	25	Did authors provide a description of the coding tree?	Supplementary material
Derivation of themes	26	Were themes identified in advance or derived from the data?	6
Software	27	What software, if applicable, was used to manage the data?	6
Participant checking	28	Did participants provide feedback on the findings?	5-6
Reporting			
Quotations presented	29	Were participant quotations presented to illustrate the themes / findings? Was each quotation identified? e.g. participant number	10-16, supplementary material
Data and findings consistent	30	Was there consistency between the data presented and the findings?	10-16, supplementary material
Clarity of major themes	31	Were major themes clearly presented in the findings?	10-16, supplementary material
Clarity of minor themes	32	Is there a description of diverse cases or discussion of minor themes?	10-16

For peer review only

Supplemental file 2

1. Patient / relative / carer topic guide.

2. Initial thematic framework

3. Table with summary of themes, sub-categories and verbatim quotes with participant numbers

For peer review only

Interview topic guide: Patient representative

Introduction:

- We value your opinions/what you think about these questions; there's not necessarily a right or wrong answer; it's not a test.

Examples of general probes that may be used

- Tell me more about that.
- Why do you think that?
- Have you got any examples?

If unable to answer open question:

- Some people have said this … [e.g. a known barrier from the literature if not already mentioned – see below for specific examples] …what do you think?

If participant unable to define early mobilisation:

- We are defining early mobilisation as something the patient does with 'their own muscle strength and control' including activities such as:
  - Moving in bed
  - Exercises
  - Sitting on the edge of the bed,
  - Standing
  - Marching on the spot,
  - Transferring from bed to chair
  - Walking…

…all whilst patients are on intensive care.

For peer review only - http://bmjopen.bmj.com/site/about/guidelines.xhtml

Interview questions

General questions:

1. Please tell me about your experience on the ICU.

2. Please tell me about physiotherapy and mobilisation (moving around and getting up and out of bed) on the ICU.
    a. What you would think of as early mobilisation?
    b. What early mobilisation did you do? How often did you do it?
    c. How was it decided that it was the right time for you to start mobilising on the ICU?

3. Do you think that early mobilisation was carried out enough?

Focused questions:

4. When you were asked if you wanted to mobilise, did you have any concerns over risks or problems that might occur?
   Potential question-specific prompts: not being well enough; fall; lines and drains/breathing tube falling out.

5. Was there anything that stopped or delayed you from mobilising on the ICU?

   Potential question-specific prompts: Feeling too unwell; lines and tubes; not enough staff.

6. What kind of things stopped mobilisation from happening more often on the ICU you were on?

For peer review only

Potential question-specific prompts: Not a priority; team did not work together; team did not have enough teaching.

7. Why do you think the staff on you ICU wanted you to mobilise and what do you think the benefits were?

   Example of potential question-specific prompts: Physical benefits; leave ICU sooner, prevent long-term complications.

8. In your experience, what things helped you to mobilise on the ICU?

   Potential question-specific prompts: Different professions working together; feeling well.

9. What kind of things do you think could be improved or changed to help mobilisation to happen more often on your ICU and to overcome some of the problems you mentioned earlier?

   Potential question-specific prompts: Better team communication; staff getting more teaching.

10. This question will explore other areas that previous interviews have brought up as important: e.g. other people have mentioned this… what do you think?

11. Is there anything else you'd like to say about what stops early mobilisation on the ICU and what could make it happen more often?

For peer review only

Initial thematic framework.

BARRIERS

1. Perceived risk of mobilising certain patients [SAFETY/RISK]

1.1 Airway and attachments

1.2 Patient instability

1.3 Patient type

1.4 Patient cognitive state

1.5 Patient medical status

1.6 Clinicians' perception of readiness to mobilise

1.7 Other

2 Patient's or their family member's reluctance for mobilisation

2.1 Clinician opinion of patient's or their family member's reluctance for mobilisation

2.2 Patients not feeling ready or motivated for mobilisation

2.3 Poor communication from clinicians

2.4 Aspects of the ICU environment not promoting mobilisation to patients

2.5 Other

3 Team working and unit culture/staff experience/ resources

3.1 Culture/Lesser priority

3.2 Roles and responsibilities

3.3 Lack of leadership

3.4 Staff experience

3.5 Lack of knowledge

3.6 Lack of resources

3.7 Logistics/ Other interventions

3.8 Nurse environment e.g. HDU/toilet

3.9 Other

FACILITATORS

4 Practical changes to how mobilisation was carried out.

4.1 Patient and family engagement

4.2 Mob treatment specific/functional rehabilitation

4.3 Use of protocols to facilitate clinical implementation of mobilisation

4.4 Patient opinion on how clinicians should communicate with them.

4.5 Specific patient motivators

For peer review only

4.6 Equipment/environment

4.7 Other changes/optimal practice

5. Improvements in team working and culture/clinician specific

5.1 Leadership

5.2 Team Communication

5.3 Experienced staff

5.4 Improved staffing/resources

5.5 Other team working

5.6 Prioritise

5.7 Education

5.8 Other culture change

6. Patient characteristics that made it easier to mobilise

6.1 Pre-morbid/general characteristics status

6.2 Acute/admission-related status

6.3 Other

7. Risks, benefits and other

7.1 Risks

7.2 Benefits

7.3 Other/irrelevant

For peer review only

Table: *Summary of themes and sub-categories*

Themes	Sub-categories	Quote number	Participant quote
1. Safety and physiological concerns	1.1 Airway, lines and attachments	1	*"I can't think who, they said an intensive care patient looks like little spiders in a web, and I agree with it. Like literally they have got tubes and attachments out everywhere." (Therapist 1)*
	1.2 Particular patient groups	2	*"…the types of patients we have have multiple and complex injuries, they're not straightforward patients to mobilise anyway…" (Therapist 3)*
	1.3 Physiological instability or dependence on organ support	3<br>4<br>5	*"…it's mainly blood pressure related for me, or their resp[iratory] rate. If I don't think they're going to tolerate mobilising, and if it's going to cause more harm than good." (Therapist 2)*<br>*"So anybody who's on an inotrope vasopressor is, as far as I'm concerned, not safe to be mobilised… they're more likely to have a postural hypertension that would result in injury to them." (Doctor 2)*<br>*"…whilst in itself [vasoactive drugs are] often not a reason to prevent ongoing rehab especially in junior staff it's a significant source of anxiety of doubling or trebling the dose of a medicine to keep your blood pressure up, without some form of kind of very clear guidance and encouragement that this is okay and it will return to normal following [rehabilitation] treatment." (Nurse 5)*
	1.4 Patient's ability to actively participate	6<br>7<br>8	*"And after that incident I think that was the first time I actually cried, because it hit me that "Yes, the nurses are right, I am not able to just get up and move like I would if I had been healthy," you know, so that was very traumatic for me…" (Patient 11).*<br>*"I think I would have felt very vulnerable anyhow, [be]cause suddenly you are just weak as a baby." (Patient 7)*<br>*"Their cognitive state is a massive thing as well. How alert are they if they've only just been woken up from sedation or if they've had a neurological event or, you know, whatever reason, that could affect their cognition." (Therapist 6)*
	1.5 Clinician perception of readiness to begin rehabilitation	9<br>10	*"…the perception, [clinicians] might think that, because this patient is dependent on a particular type of organ support, this patient is not suitable for mobilisation. So these boundaries and barriers needs to be broken." (Doctor 3)*<br>*"I think it's probably the fear of the unwell patient, you know, we, they're in ITU therefore they must be the most unwell people in the hospital. And I think it's that kind of mentality and the fact we attach them to fifteen hundred things…" (Therapist 7)*

For peer review only

		11	*"…happy to cause no harm, or kind of, and no perceived harm by not mobilising someone but actively getting up and causing harm is a, always going to be a significant anxiety for staff…" (Nurse 5)*
		12	*"We love keeping the numbers normal, we love the sense of security that we maintain as normal physiology as we can, so that is why, a junior nurse would be more worried if she gets any change in the patient's state after mobilisation…" (Doctor 1)*
		13	*"…intensive care doctors and nurses may also be quite, well, I shouldn't say "quite" but there is a part in us that is controlling the situation and so, you know, trying to mobilise the patient may also be a bit of a paradigm shift in our own mind of, you know, this complete control over the situation and over this patient." (Doctor 4)*
		14	*"…so we kind of reset our expectations about normality and that is doing some sort of exercise when they ambulate because it is – it is to a critically ill patient it is an exercise, that we may see some events happen and as long as it is the range of acceptance, we can just modify our targets, and continue to mobilise." (Doctor 1)*
2. Patient participation and engagement	2.1 Patient reluctance to participate in rehabilitation	15	*"...well I'm in ICU... you're having intensive care, don't rock the boat by making things worse by trying to get out of bed." (Patient 8)*
		16	*"…there were times when I simply didn't want to do it… Depression, … lack of energy, lack of spirits really …" (Patient 7)*
	2.2 Communication between patients and clinicians	17	*"I would say to any nurse or any staff working in ICU… keep up that reassurance with patients because it's quite a scary experience…" (Patient 8)*
		18	*"[The consultant] pushed me beyond what I mentally thought was physically possible. I didn't believe that I could do that and of course, perhaps it's the nature of my personality, but I responded to that. Others may not have responded to that, I can't say." (Patient 3)*
	2.3 Patient engagement in planning rehabilitation	19	*"So, alongside that, we've also made like goal setting sheets that can go up by the patient's bed, so then when they sit up, when they sit upright in bed, they can see them. I draw a smiley face when they've completed one…" (Therapist 2)*
	2.4 Including activities meaningful to patients	20	*"Looking at therapy in a slightly different way and finding an activity that's meaningful to [patients], whether that's personal care or leisure activities, and through that encouraging them to… engage in that activity and then helping them to see the therapeutic value of that." (Therapist 4)*
	2.5 Identify key patient motivators	21	*"…if they can see what's in it for them, that they're gaining in dignity and all of that, they might cooperate more." (Relative 2)*

For peer review only

	2.6 The role of family	22	*"I remember the first time I took a few steps, the nurse said to me, "Well we'll do it with your husband," so my husband stood on one side and said, "We'll go for a walk with your husband," ... So it was most amazing feeling ever, you know? So everything, kind of in my head everything shut down; the nurse went away, the ward went away, it was just me and my husband going for a walk." (Patient 11)*
3. Clinician experience and knowledge	3.1 Amount of experience and support	23	*"I've had instances where it's mostly been junior people and it's terrifying. But for someone then to have a senior position helping you, that's so much better." (Nurse 2)*
	3.2 Lack of training, knowledge and skills	24	*"It doesn't happen because… we are not aware enough yet how important it is, or how much difference it could make, so it's not embedded in our thinking and in our behaviour well…" (Doctor 4)*
	3.3 Interdisciplinary team education and training	25	*"The education as well is important because you need to get people to understand what they're doing and to value it, so that they do it with passion and with skill." (Doctor 1)*
4. Teamwork	4.1 Team culture and attitudes	26	*"But a lot of it's just to do with the attitude of the individual staff member, how proactive they are and how much they believe in mobilisation as a kind of key thing" (Nurse 5)*
	4.2 Perception of roles and responsibilities	27<br>28<br>29<br>30	*"…I think the consultant's role is very important and it doesn't just include saying, "Mobilise the patient". It includes making sure that mobilisation happens and making sure that the team are, like every single member of the team is comfortable and understands the decision, and the risks related to it and understands that I am there to back them up if something happens." (Doctor 1)*<br>*"I've found that it's taken a long time for me to be accepted and for them to actually accept my opinion might be right…" (Therapist 2)*<br>*"I always felt like it was, it was very much seen it was the physio job to do anything related to moving the patient so even getting them out of bed." (Therapist 7)*<br>*"...good teamwork is really helpful, and actually a really good symbiotic relationship between the nursing staff and the therapy staff is really key." (Therapist 4)*
	4.3 Definition and delivery of rehabilitation	31<br>32<br>33	*"…mobilisation for me in ITU is hoisting somebody into a chair." (Nurse 4)*<br>*"...rehabilitation is not, you know, 20 minutes with the physio or the OT every day. Really good rehabilitation is a 24 hour approach, and that – part of that is positioning a patient in bed. Part of that is ensuring the patient gets the right nutrition as well as looking at the actual physical things that they're doing." (Therapist 4)*<br>*"...a different mentality within intensive care and to think, well actually, you know, we need to begin the rehab process all together from day one, and if a patient can be encouraged to do something they should be given the time and the opportunity to do that." (Therapist 4)*

For peer review only

	4.4 Staffing and logistics	34	*"…you start breaks at 9.30, 10.00. You finish the breaks about 12.00,1.00, so then it's not until the afternoon that people are free to help." (Nurse 2)*
5. Equipment and environment	5.1 Rehabilitation equipment	35	*"…equipment wise. You know, it's the age old problem isn't it, more of it, better ways to fix it, more money, so we've got the equipment, you know, got backup hoists." (Therapist 7)*
	5.2 ICU environment	36<br>37	*"there's just something about the environment which makes you think that you need to stay in the bed and that you shouldn't be moving around… whereas on a general ward, you don't want to be in the bed, you want to get out." (Patient 8)*<br>*"…you can see some bright lights and monitors, you can hear monitors going off, but you don't have the, "Crash, bang, wallops!" that you get in a general ward... but it's a capsule and a bubble, it's a weird feeling... "People think it's like being in a spaceship" and I thought, "That's such a good description" and that's how it did feel." (Patient 8)*
6. Risks and benefits of rehabilitation on intensive care	6.1 Clinician perception of risks	38<br>39	*"Falls, removal of lines and tubes and then causing bleeding, vasovagal episodes, it's actually a risky thing to mobilise an ITU patient, anything can go wrong…" (Nurse 3)*<br>*"I like mobilising patients. The more attachments the better… Because I like the challenge!" (Therapist 2)*
	6.2 Patient perception of risks	40	*"I never felt scared, I felt that the physiotherapist that was orchestrating the movement was sort of holding on to me to begin with and I never felt I was going to fall down…" (Patient 1)*
	6.3 Physical benefits	41	*"It might help their movement and I feel the more they mobilise the more their muscles are good. The more you make them sit out of the bed and stand they can stand on their feet better." (Nurse 1)*
	6.4 Psychological benefits	42	*"…the important thing is you sense that you're not just lying there waiting to die. ...so you are… you are… coming back to being a human being that wants to live." (Patient 7)*

For peer review only

BMJ Open

Clinician and patient perspectives on the barriers and facilitators to physical rehabilitation in intensive care: a qualitative interview study

Journal:	*BMJ Open*
Manuscript ID	bmjopen-2023-073061.R1
Article Type:	Original research
Date Submitted by the Author:	05-Sep-2023
Complete List of Authors:	Woodbridge, Huw; Imperial College Healthcare NHS Trust; Imperial College London, Department of Surgery and Cancer<br>Norton, Christine; Kings College London, Florence Nightingale Faculty of Nursing, Midwifery and Palliative Care<br>Jones, Mandy ; Brunel University London<br>Brett, Stephen; Imperial College Healthcare NHS Trust, Anaesthetics and Intensive Care; Imperial College London, Department of Surgery and Cancer<br>Alexander, Caroline; Imperial College Healthcare NHS Trust, Therapies; Imperial College London, MSk Labs<br>Gordon, Anthony; Imperial College London, Department of Surgery and Cancer; Imperial College Healthcare NHS Trust
**Primary Subject Heading**:	Intensive care
Secondary Subject Heading:	Rehabilitation medicine, Qualitative research
Keywords:	Adult intensive & critical care < INTENSIVE & CRITICAL CARE, QUALITATIVE RESEARCH, REHABILITATION MEDICINE

SCHOLARONE™
Manuscripts

I, the Submitting Author has the right to grant and does grant on behalf of all authors of the Work (as defined in the below author licence), an exclusive licence and/or a non-exclusive licence for contributions from authors who are: i) UK Crown employees; ii) where BMJ has agreed a CC-BY licence shall apply, and/or iii) in accordance with the terms applicable for US Federal Government officers or employees acting as part of their official duties; on a worldwide, perpetual, irrevocable, royalty-free basis to BMJ Publishing Group Ltd ("BMJ") its licensees and where the relevant Journal is co-owned by BMJ to the co-owners of the Journal, to publish the Work in this journal and any other BMJ products and to exploit all rights, as set out in our licence.

The Submitting Author accepts and understands that any supply made under these terms is made by BMJ to the Submitting Author unless you are acting as an employee on behalf of your employer or a postgraduate student of an affiliated institution which is paying any applicable article publishing charge ("APC") for Open Access articles. Where the Submitting Author wishes to make the Work available on an Open Access basis (and intends to pay the relevant APC), the terms of reuse of such Open Access shall be governed by a Creative Commons licence – details of these licences and which Creative Commons licence will apply to this Work are set out in our licence referred to above.

Other than as permitted in any relevant BMJ Author's Self Archiving Policies, I confirm this Work has not been accepted for publication elsewhere, is not being considered for publication elsewhere and does not duplicate material already published. I confirm all authors consent to publication of this Work and authorise the granting of this licence.

For peer review only

Clinician and patient perspectives on the barriers and facilitators to physical rehabilitation in intensive care: a qualitative interview study.

Authors

Huw R. Woodbridge*

- Imperial College Healthcare NHS Trust, London, UK; Imperial College London, London, UK.

Christine Norton

- King's College London, London, UK.

Mandy Jones

- Brunel University London, London, UK.

Stephen J. Brett

- Imperial College Healthcare NHS Trust, London, UK; Imperial College London, London, UK.

Caroline M. Alexander

- Imperial College Healthcare NHS Trust, London, UK; Imperial College London, London, UK.

Anthony C. Gordon

- Imperial College London, London, UK; Imperial College Healthcare NHS Trust, London, UK.

*Corresponding author: Physiotherapy Department, St Mary's Hospital, Praed Street, London W2 1NY. Email: h.woodbridge@imperial.ac.uk Telephone: 0203 312 1794.

Keywords: Adult intensive & critical care; qualitative research; rehabilitation medicine.

Word count: 5718

For peer review only

Abstract

Objectives

To explore patient, relative/carer and clinician perceptions of barriers to early physical rehabilitation in intensive care units (ICU) within an associated group of hospitals in the United Kingdom (UK) and how they can be overcome.

Design

Qualitative study using semi-structured interviews and thematic framework analysis.

Setting

Four ICUs over three hospital sites in London, UK.

Participants

Former ICU patients or their relatives/carers with personal experience of ICU rehabilitation. ICU clinicians, including doctors, nurses, physiotherapists and occupational therapists, involved in the delivery of physical rehabilitation or decisions over its initiation.

Interventions

Nil

Primary and secondary outcomes measures

Views and experiences on the barriers and facilitators to ICU physical rehabilitation.

Results

Interviews were carried out with 11 former patients, 3 family members and 16 clinicians. The themes generated related to: safety and physiological concerns; patient participation and engagement; clinician experience and knowledge; teamwork; equipment and environment; and risks and benefits of rehabilitation in intensive care. The overarching theme for overcoming barriers was a change in working model from ICU clinicians having separate responsibilities (a multidisciplinary approach) to one where all parties have a

shared aim of providing patient-centred ICU physical rehabilitation (an interdisciplinary approach).

Conclusions

The results have revealed barriers that can be modified to improve rehabilitation delivery in an ICU. Interdisciplinary working could overcome many of these barriers to optimise recovery from critical illness.

Strengths and limitations of this study

- This study explored a range of perspectives on the barriers to ICU rehabilitation (including clinicians and services users), thus eliciting in depth information to reveal a breadth of experiences of barriers and facilitators.
- Thematic framework analysis was used which enables a systematic approach to organising data, facilitating in-depth exploration of the range of views within themes and between participant groups.
- Patient and family recall of their experiences may have been impacted by the time from intensive care admission to interview, however interviews took place at the first follow up opportunity to minimise this effect.
- Efforts were made to gain a range of perspectives using purposive sampling; however, fewer family members or carers took part in this study than former ICU patients.

Introduction

The importance of physical rehabilitation of critically ill patients has been recognised because of the prevalence of acute muscle weakness and wasting [1-3], and longer-term substantial physical disability measured in this patient group [2 4 5]. Physical rehabilitation consists of physical activity interventions (typically mobilising in or away from the bed) that are begun once a patient has reached physiological stability [6-8]. Beginning physical rehabilitation at an appropriate dose whilst patients are still in an intensive care unit (ICU)

For peer review only

can improve physical function whilst in hospital and expedite discharge [9], although implementing rehabilitation at a higher dose is not necessarily beneficial [10]. However, when measured, there is concern that the actual amount of formal physical rehabilitation delivered and patient participation in exercise whilst in intensive care are low [11-17].

Studies have previously measured the barriers to implementing rehabilitation, the majority of which use a quantitative approach [18]. However, a qualitative approach is better-suited to exploring interpersonal relations [19] and therefore potential barriers relating to team working and patient interactions. Where a qualitative approach has been used, issues of communication and differences in opinion between clinicians [20-25] and difficulty in providing rehabilitation in an environment where demands on staff and patient time change quickly have been highlighted [23 26 27]. However, the lack of rehabilitation in intensive care continues despite this current understanding of the barriers. Importantly, there is a lack of in-depth knowledge of barriers in a United Kingdom (UK) setting, which includes views of multiple stakeholders such as ICU clinicians from different professions involved in implementing rehabilitation, as well as patients and family members [22 28-30].

The objective of this study therefore, was to explore service user (patients or their relatives/carers) and clinician perceptions of barriers to early physical rehabilitation in ICUs within an associated group of hospitals in the UK and how they can be overcome.

Methods

Research design

A qualitative study using semi-structured interviews was conducted based on the approach recommended by the National Centre for Social Research. This is based upon critical realism and interpretivism using the framework approach to analysis [31]. Ethical approval was gained from the London – Bloomsbury Research Ethics Committee (17/LO/0362) and written, informed consent was gained from all participants. This study is reported in line with the consolidated criteria for reporting qualitative research [32] (supplemental file 1). The study was managed by a research steering group consisting of four researchers with

For peer review only

subject and methods experience (HRW, CN, CMA, ACG) and two patient representatives who were former ICU patients approached through a national patient support group.

Setting and participants

The study was based at a UK National Health Service (NHS) hospital trust in London, which has four ICUs for adult patients across three hospital sites, each of which has different referring specialities. Purposive sampling [33 34] was used to recruit a range of service users (former ICU patients and their family members/carers) and the hospital's ICU clinicians from the different ICU settings, from different professional groups with a range of experience levels.

Eligibility criteria and recruitment strategies

Clinicians were included if they were doctors (senior trainees [registrars/fellows] or consultants), nurses, occupational therapists or physiotherapists with at least two months of ICU experience and who had experience of rehabilitation treatments or deciding when they should be initiated. Clinicians were approached via adverts in meetings, posted in staff areas or via general group or more targeted emails. Former ICU patients and family members were included if they had any personal experience of physical rehabilitation whilst in ICU. Participants were excluded if they could not attend an in-person interview, if they felt unable to participate in English, if they were less than 18 years of age or unable to give informed consent. Patients or their relatives/carers were approached via local ICU patient support groups and follow up clinics.

Data saturation

Estimates were that 30 participants would be required to gain a sufficient range of perspectives, with sampling ending once apparent data saturation had been reached. Data saturation was defined a priori as when no new themes of barriers and facilitators were evident from interviews, as decided by the research steering group. During data collection, when the interviewer felt no new themes were being discussed, the latest version of the initial thematic framework was shown to the final clinician and patient participant as a sense check to see whether they could identify any additional themes that had been missed. Following this, the initial thematic framework was reviewed by clinical colleagues from all

For peer review only

four professional groups included in the study, as well as the research steering group members who were former ICU patients, to discuss if any obvious themes were missing.

Topic guide development

A semi-structured interview topic guide was developed (supplemental file 2), designed by the research steering group (which included the input of former ICU patients) and was formatted based on typical qualitative interview procedures [35-38]. It consisted of 11 main open questions that were designed to address different aspects of the research objective (such as barriers and risk), some of which were derived from previous studies[20 27 39]. The interview was piloted with both clinicians and former patients to ensure questions were clear and fit for purpose. No modifications were required to the topic guide in response to testing.

Interview procedures

Interviews took place at the hospital site in person and only included the interviewer and the individual participant. Each participant took part in one semi-structured interview. Before the interview began, participants were asked for demographic data then the interview proceeded. The format of the interview was a conversation, where wording was not fixed and prompts were used to gain greater depth of understanding of participant views and experiences[35]. Participants were asked to define physical rehabilitation themselves, however, the study was designed based on rehabilitation consisting of mobilisation treatments ranging from exercises and movement in the bed, to mobilising out of bed and walking [8]. Participants were informed of this if they had difficulty defining rehabilitation or if their definition was markedly different from how the study conceptualised rehabilitation. Each interview was recorded and then transcribed verbatim. Transcripts were not returned to participants for review in line with current thinking about usefulness of this approach [40].

Reflexivity

All interviews were carried out by one interviewer (HRW) who is a male physiotherapist, working full-time on the research study as part of work towards a doctorate, with training in qualitative research methods. The interviewer had previous clinical experience at several of

For peer review only

the ICUs that were settings for this study, including working alongside some of the clinician participants, but not the patients or their relatives/carers. The researcher therefore had previous experiences of barriers and facilitators of rehabilitation in the study setting. These influences were taken into account using a reflexive diary before and after data collection, which was then considered during the analysis process.

Data analysis

Thematic framework analysis [41 42] was used to produce themes based on the interview transcript data. This involves drawing up an initial list of themes that summarised all the interview data (supplemental file 2). Data were then arranged in a framework table which structured what each participant had said about each initial theme in an easily accessible form. This facilitated the production of a final set of themes and subthemes and comparison of how these vary between groups of participants. Analysis was facilitated by the use of NVivo 11 software (QSR International) and carried out by the first author (HRW). A second researcher (MJ) reviewed 10% of interview transcripts and confirmed that they matched the initial set of themes. At several stages during the analysis process, the research steering group met to review the data, discuss uncertainties over formation of themes and as a check on the process. Descriptive statistics were used to summarise demographic data using IBM SPSS Statistics 25. Continuous data were tested for normality using the Shapiro-Wilk test and non-normally distributed data described using median and interquartile range, and normally distributed data described using mean and standard deviation.

Patient and public involvement

Former ICU patients were members of the research steering group. These patient representatives edited the wording of recruitment materials and inputted into the design of the topic guide. They also assisted the interviewer (HRW) to practice interview technique and were involved in reviewing the initial thematic framework, as part of data saturation checks. These patient representatives did not participate or contribute data to the study itself.

For peer review only

Results

Recruited participants included 16 clinicians, from a range of professions, with a range of experience in different settings (Table 1). Eleven former ICU patients and three family members/caregivers participated (Table 2), including substantial patient experience of ICU rehabilitation (mean patient participant length of ICU stay: 15 days, standard deviation ± 10.8 days). Initially, 53 potential participants expressed interest in taking part, of whom 30 were recruited before data saturation was achieved. Five declined or were not available for interview, three did not respond further after initial contact, data saturation was achieved before four were recruited and 11 were not recruited as others were chosen instead to gain a greater range of views, as per the purposive sampling strategy. Interviews lasted for a mean 43 minutes (standard deviation ± 11 minutes).

For peer review only

Table 1: Clinician participant demographics	
	**Clinicians (N=16)**
Age, mean (± SD)	34 (8.6)
Gender: female, n (%)	12 (75)
Profession, n (%)	
Doctor	4 (25)
Nurse	5 (31)
Therapist (physiotherapist or occupational therapist)	7 (44)
Seniority, n (%)	
Team leader	9 (56)
Senior clinician	6 (38)
Junior clinician	1 (6)
Number of years of ICU experience, median (IQR)	6 (1-15)
Number of years of clinical healthcare experience, mean (± SD)	11 (8)
Place of work*, n (%)	
Intensive care 1	5 (31)
Intensive care 2	6 (38)
Intensive care 3	5 (31)
Intensive care 4	5 (31)
Involvement in physical rehabilitation, n (%)	
Participating in the decision over whether a patient is stable enough to mobilise.	16 (100)
Leading rehabilitation treatment	10 (63)
Assisting with rehabilitation treatment	12 (75)

SD= standard deviation; IQR=interquartile range. *Some clinicians work on more than one intensive care unit.

For peer review only

Table 2: Patient and caregiver participant demographics	
	**Former ICU patients and caregivers (N=14)**
Service user participants:	
Patients, n (%)	11 (79)
Caregivers, n (%)	3 (21)
Age, mean (±SD)	65 (10.7)
Male, n (%)	10 (71)
Patient ICU length of stay in days* (n=11), mean (±SD)	15 (10.8)
Patient stated reason for admission (n=11), n (%)	
Aortic dissection	1 (9)
Cardiac arrest	1 (9)
Gastrointestinal	1 (9)
Organ failure	1 (9)
Septic shock	1 (9)
Surgery	5 (45)
Trauma	1 (9)
Site where ICU was experienced, n (%)	
Intensive care 1	2 (14)
Intensive care 2	11 (79)
Intensive care 3	0 (0)
Intensive care 4	1 (7)
Highest level of physical rehabilitation experienced, n (%)	
Moving in bed	2 (14)
Sitting in a chair	6 (43)
Walking	6 (43)
SD= standard deviation. *Two participants reported their length of stay as approximate.	

The study themes and subthemes are described in detail below. The supplementary material illustrates these themes and subthemes with verbatim quotes from participants, with participant numbers (see supplemental file 2 to link quote numbers with data).

1. *Safety and physiological concerns*

Clinician and patient participant concerns over the safety of rehabilitation were reported as a barrier to rehabilitation. This included the risk of dislodging lines and attachments (such as ventilator tubing and femoral lines (quote 1). However, some participants, who were mostly clinicians, did not perceive this as a barrier, if careful planning and also organisation of the bed space environment was carried out. For example, avoiding the use of femoral vascular catheters as access for haemofiltration or planning breaks in haemofiltration could enable rehabilitation. Endotracheal tubes or airways that had been difficult to insert, were also

For peer review only

cited as barriers, with the difficulty of titrating sedation for a balance between tube tolerance and patient alertness cited as one explanation by a clinician.

Clinician participants identified particular patient groups with barriers to rehabilitation because they felt they were at an increased risk or they presented additional logistical challenges, such as those with multiple traumatic injuries (quote 2). They suggested that patients admitted after surgery could have certain surgical precautions which presented logistical issues contacting different teams to gain clarity over safety of rehabilitation. Despite this, patients in ICU after elective surgery could have received pre-operative education or pre-planned rehabilitation programmes, both facilitating rehabilitation post-operatively.

Physiological instability, such as instances of respiratory distress or cardiovascular instability were reported as preventing rehabilitation treatments by participants who were mostly clinicians.

> *"…it's mainly blood pressure related for me, or their resp[iratory] rate. If I don't think they're going to tolerate mobilising, and if it's going to cause more harm than good." (Therapist 2, quote 3)*

Dependence on organ support, such as the amount of respiratory support or vasoactive drugs were also identified as barriers. Clinician opinion ranged from perceiving patients receiving vasoactive drugs as a contraindication to rehabilitation (quote 4), to others who considered rehabilitation possible if a low or weaning dose was used or if the patient was less severely unwell, for example if vasoactive drugs were being used for epidural-induced hypotension. Risk relating to hypotension during rehabilitation was suggested by a clinician to relate to anxiety from junior staff about managing vasoactive drugs during mobilisation (quote 5). Some clinician participants suggested potential organ support barriers should be discussed with the ICU doctors and also advocated actively sedating patients less.

Patient participants sometimes reported feeling too unwell to actively participate in rehabilitation. Some patients reported profound feelings of weakness, making their bodies feel 'like a lead weight', which came as a surprise when they first tried to get up and was linked with feelings of vulnerability (quotes 6 and 7). These participants did then identify a

For peer review only

time in their recovery where these symptoms subsided to the point where they could then participate.

Additionally, level of alertness, confusion and agitation, cognitive impairments and personality disorders were all cited as barriers by clinicians (quote 8). Some patients and relatives recalled experiences of delirium and hallucinations as profound influences on their recovery in general.

The difference between clinicians' perception of safety and a patient's readiness to begin rehabilitation was expressed as a barrier by clinician participants (quote 9). Some explanations included clinician fear of the unwell patient and the risk of perceived harm which caused anxiety for some (quote 10).

> *"…happy to cause no harm, or kind of, and no perceived harm by not mobilising someone but actively getting up and causing harm is a, always going to be a significant anxiety for staff…" (Nurse 5, quote 11)*

This was linked to clinician need for control over the physiological numbers, potentially leading to a reluctance to reduce that control by moving a patient out of bed (quote 12). One doctor suggested that a paradigm shift was required to address this barrier (quote 13). Another doctor said they modified targets for acceptable changes in physiological observations (such as blood pressure), to reassure other clinicians that mobility was still safe (quote 14).

2. *Patient participation and engagement*

Clinician participants reported experience of patients who may be reluctant to participate in rehabilitation. When asked about this theme, patient participant responses ranged from reporting enthusiastic engagement in rehabilitation, to not wishing to mobilise out of bed. Reasons cited for their reluctance included not wanting to do something perceived as potentially worsening their condition (quote 15). Furthermore, a lack of incentive or motivation to engage was discussed, as well as a feeling of weakness, which some found difficult to accept.

For peer review only

"…there were times when I simply didn't want to do it… Depression, … lack of energy, lack of spirits really …" (Patient 7, quote 16)

Suboptimal communication between patients and clinicians was felt to be a barrier to rehabilitation by some patient and clinician participants. Suggested reasons included the little time spent by clinicians discussing rehabilitation, difficulty communicating rehabilitation goals and some sometimes showed a lack of empathy. Suggested ways of overcoming these issues included maximising a patient's ability to communicate, giving more reassurance, building up trust, showing kindness and helping patients to feel safe (quote 17). Patients valued humour from staff and felt rapport was aided by staff continuity. Patients and relatives recommended that when a patient was reluctant to mobilise, an encouraging and diplomatic approach balanced with assertiveness from clinicians to "push" patients (quote 18).

Some patient participants recommended that strategies to improve patient engagement in rehabilitation should always be patient-specific. Other suggestions, mostly from clinicians, included promoting sleep at night, involving patients in planning a rehabilitation timetable, goal setting and using outcome measures to demonstrate progress (quote 19). Furthermore, education for patients and relatives at the appropriate time, around the importance of rehabilitation was suggested.

Further facilitators suggested by patients and clinicians included the use of meaningful activities and identifying key patient motivators. The importance of tailoring rehabilitation to include activities meaningful to patients (such as functional tasks and personal care activities based on previous interests) were identified to facilitate engagement within a context more readily understood by patients.

"Looking at therapy in a slightly different way and finding an activity that's meaningful to [patients], whether that's personal care or leisure activities, and through that encouraging them to… engage in that activity and then helping them to see the therapeutic value of that." (Therapist 4, quote 20)

Recognising key patient motivators such as gaining independence and dignity by being able to do more for themselves was also suggested (quote 21). Patients reported being

For peer review only

motivated through their improvement during rehabilitation sessions, almost as a proxy for improvement from critical illness. Patient qualities of resilience, determination and a positive mental attitude were reported as a facilitator by patients themselves.

The role of family was discussed as both a barrier and facilitator. Instances were reported by some clinician participants where relatives could be reluctant for patient participation in rehabilitation. When this was discussed with patient and relative participants, responses ranged from an understanding of why this happens, to a strong disbelief that this could be the case. The role of family in encouraging patients was discussed, with some highlighting how they were motivated to improve mobility to help their family member feel better (quote 22).

3. Clinician experience and knowledge

Clinician participants discussed the experience and knowledge of those carrying out rehabilitation. A lack of experience, confidence and senior support were cited as barriers (quote 23). However, some therapists also proposed those clinicians with more experience could pose a barrier. They suggested some more experienced nurses may perceive rehabilitation as outside of their role or may have spent more time in an environment where rehabilitation was not a priority. Opinions over experience as a facilitator also varied. Some emphasised that a team with the right skill mix (including adequate senior support) was important, with a nurse suggesting having more confident staff freed up time for rehabilitation. However, some therapists reported that more inexperienced nurses could be a facilitator as they have received recent training in rehabilitation. One therapist cited enthusiasm as being more important than experience to facilitate rehabilitation.

A lack of training and knowledge, including about the importance of rehabilitation, organisation and planning of sessions and therapeutic manual handling were suggested as important factors by clinicians.

> *"It doesn't happen because… we are not aware enough yet how important it is, or how much difference it could make, so it's not embedded in our thinking and in our behaviour…" (Doctor 4, quote 24)*

For peer review only

A popular strategy suggested by clinicians to address these barriers was through education and training for the ICU interdisciplinary team, such as through study days and experiential learning (quote 25). Additionally, the use of a rehabilitation policy and guidelines to drive implementation and aid less experienced clinicians know when to begin rehabilitation was discussed.

4. *Teamwork*

Discussion of teamwork covered team culture, clinician roles, rehabilitation definitions and logistics. A lack of a rehabilitation culture leading to some staff having a less proactive attitude to rehabilitation delivery was discussed.

> *"But a lot of it's just to do with the attitude of the individual staff member, how proactive they are and how much they believe in mobilisation as a kind of key thing" (Nurse 5, quote 26)*

One explanatory factor was a lack of medical leadership. Participants (mostly clinicians) suggested promoting a culture where an interdisciplinary team works together to promote rehabilitation as routine and important, would facilitate implementation. A less hierarchical culture would encourage proactive team planning and problem solving, with medical leadership again emphasised as key (quote 27).

Another key barrier to rehabilitation discussed by clinicians, was differences in opinion between professions over roles and responsibilities (quote 28). Some reported that rehabilitation was perceived as only a therapist's job (quote 29). Therapists reported that there could be a lack of understanding of their role or their other responsibilities, for example, covering other clinical areas in addition to the ICU. To overcome this, clinicians suggested promoting teamwork where separate responsibilities were acknowledged and there was a willingness to crossover professional roles, with therapists empowering nurses to facilitate rehabilitation (quote 30).

Differences in opinions over roles and responsibilities were impacted upon by variation in how rehabilitation was defined and delivered. This in itself may explain some of the difficulty in promoting a proactive rehabilitation culture. Clinicians sometimes limited their

For peer review only

definition of rehabilitation to a patient sitting out in a chair (quote 31). Conversely, occupational therapist (OT) participants widened the concept of rehabilitation to encompass a twenty-four hour interdisciplinary approach utilising functional tasks.

> *"...rehabilitation is not, you know, 20 minutes with the physio or the OT every day. Really good rehabilitation is a 24 hour approach, and that – part of that is positioning a patient in bed. Part of that is ensuring the patient gets the right nutrition as well as looking at the actual physical things that they're doing." (Therapist 4, quote 32)*

This may increase patient engagement and interdisciplinary involvement, by helping staff to incorporate more rehabilitation activities during the course of their normal duties, for example during personal care activities (quote 33).

Finally, lack of staff and logistical difficulties in implementing rehabilitation were suggested as barriers by clinicians and patients. Greater investment in staffing and utilisation of healthcare support workers was suggested to address this. Logistical concerns covered the number of staff required and the duration of a rehabilitation session in competition with other unit procedures. Logistical barriers also concerned a difficulty in timing around nurses' rest breaks and staffing ratios (quote 34). Within the study ICUs, once a patient's illness severity decreased to a certain level, the nursing staffing ratio fell from one nurse to one patient to one nurse to two patients, coinciding with a potential increase in readiness for rehabilitation. Potential strategies to address these concerns include proactive planning of sessions, for example during morning team briefings. Additionally, a change to working patterns to build in more time for rehabilitation to occur was suggested.

For peer review only

5. *Equipment and environment*

A lack of working specialist rehabilitation equipment was highlighted as a barrier by clinician participants (quote 35). Clinicians advocated greater investment and suggested the whole team to take ownership of ensuring equipment was fixed or to find funding sources for equipment replacement. Environmental concerns raised by patients and clinicians firstly covered practical limitations such as space to move rehabilitation equipment around the

bedspace. Furthermore, a patient highlighted the nature of the ICU environment itself did not encourage them to move out of bed (quote 36).

> *"...you can see some bright lights and monitors, you can hear monitors going off, but you don't have the, "Crash, bang, wallops!" that you get in a general ward... but it's a capsule and a bubble, it's a weird feeling... "People think it's like being in a spaceship" and I thought, "That's such a good description" and that's how it did feel." (Patient 8, quote 37)*

6. Risks and benefits of rehabilitation in intensive care

Opinions over risks and benefits were explored, which closely related to safety, knowledge and attitude towards rehabilitation. Clinician ideas about risks resembled the safety issues from theme one, however, this did not necessarily mean a reluctance to mobilise (quotes 38 and 39). Most patients and relatives reported they had not worried about the risks of mobilising whilst in an ICU (quote 40), although some had experienced things such as dizziness and one reported passing out. Considering benefits reported by clinicians and patients, physical benefits of rehabilitation focused on the acute impact of improving physical function, including in preparation for recovery on the wards (quote 41). Suggested psychological benefits for the patient included helping mood and wellbeing and restoring a sense of dignity.

> *"...the important thing is you sense that you're not just lying there waiting to die. ...so you are… you are… coming back to being a human being that wants to live." (Patient 7, quote 42)*

Finally, several clinicians reported how a benefit of patient rehabilitation was the encouragement and sense of achievement it provided for staff.

The overarching theme for how to overcome barriers to physical ICU rehabilitation related to moving from a multidisciplinary approach where different professions work together but have separate responsibilities; towards a patient-centred, interdisciplinary team approach. This was where all parties have a shared aim of providing physical rehabilitation (quotes 27 and 30). This can facilitate clinicians working together to develop a shared understanding of

For peer review only

the definition of rehabilitation, so patients can participate in activities that are more meaningful. Furthermore, an agreement can be developed among the team, about the benefits and risks, the optimum way to deliver rehabilitation and when it is safe to start. This can then help different professions to collaborate to help to overcome barriers related to team working and to improve the ICU environment.

Discussion

This study has provided an in-depth exploration of the views of multi-professional ICU clinicians and was strengthened by including former ICU patients and their relatives, adding to the knowledge of overcoming barriers to ICU physical rehabilitation. Primarily this is suggested to be through a change in approach to team working, from a multidisciplinary to an interdisciplinary and patient-centred approach. This means moving from a multidisciplinary way of working where a team is made up of different professions working on their distinct priorities [43-45], to an interdisciplinary approach where a team of different professions work together with ICU rehabilitation a priority for all. This therefore emphasises a shift from rehabilitation primarily being the focus of therapy staff, to one where all team members have joint accountability and identify this as a key aspect to their work, contributing in overlapping ways but also in ways relevant to their professional skills and knowledge [46 47]. This change in perspective could facilitate a change in opinion over the definition and delivery of rehabilitation towards an interdisciplinary, 24-hour approach that includes activities meaningful to patients to facilitate engagement. An interdisciplinary working model has previously been used to facilitate more efficient and effective care during critical illness and in general rehabilitation delivery. Reported outcomes have included more coordinated inter-professional working and enhanced delivery of appropriate patient care [44 48 49].

It is interesting to see that several of the themes of barriers and facilitators to ICU rehabilitation are similar to previous qualitative studies. This includes themes of safety [23 24 27 28 50], patient engagement [29], knowledge and experience [21-26] and team work [20 22-25 27-29 50]. Patient reports of experiencing feelings of weakness and vulnerability in this present study have also previously been identified [30 51] where their vulnerability

For peer review only

may be explained, at least in part, by patients adjusting to being critically ill whilst having little or no memory of their deterioration into critical illness [52]. In our study, clinicians expressed differences in opinion over roles and responsibilities towards rehabilitation as well as safety concerns for initiating treatment. Staff confidence in rehabilitation provision may contribute towards differences in viewpoints and engagement, particularly towards opinions on readiness of a patient to begin rehabilitation. This may be partially explained through differences in personality traits between those more or less able to make pragmatic concessions to adjust to the limitations of the working environment to ensure reasonable care is delivered and to tolerate greater variability in acceptable target physiological observations[53]. This would therefore represent an important factor to address with staff when overcoming barriers to rehabilitation, for example, to achieve the paradigm shift suggested by some participants to enable clinicians to address anxiety in relation to control over physiological parameters.

This study has added to previous knowledge in several ways. Interestingly and perhaps surprisingly, less clinical experience was highlighted as a potential facilitator of rehabilitation. Some therapists reported that more inexperienced nurses have received recent training in rehabilitation and one therapist cited enthusiasm as more important than experience. Additionally, perceptions of the content of rehabilitation were notable. Some viewed rehabilitation as being limited to sitting in a chair. This can contribute to a limited scope of rehabilitation practice [26] and may have contributed to the lack of rehabilitation culture reported by some participants in this study. The occupational therapists emphasised the inclusion of personal care activities as part of ICU rehabilitation delivered at any point in the day by any profession, to facilitate a more positive rehabilitation culture. This is supported by Laerkner, et al. [54] who compared the views of nurses and patients in Denmark, and found nurses recommended incorporating familiar activities into rehabilitation and patients emphasised the importance of empathy and compromise from clinicians. Whilst patients in this present study agreed with Laerkner's recommendations of clinician-patient communication, they also emphasised that at times, a more assertive approach from clinicians in encouraging rehabilitation is desirable.

The findings from this study focus us to create a patient-centred interdisciplinary approach to rehabilitation. This involves considering how clinicians communicate with patients and

broadening the definition of rehabilitation to include functional tasks that are meaningful to patients. Furthermore, broadening delivery of rehabilitation to a 24-hour holistic approach that includes family members[55-57], with a focus on prioritising patient-reported motivators of independence and dignity and to progress back towards normality. Facilitating this change in a multifaceted ICU environment would benefit from using implementation and improvement science methodology, where co-design by ICU clinicians from different professions, as well as service users can be employed. This change in practise should be evaluated not only in terms of whether it improves rehabilitation delivery without impacting patient safety, but also in terms of how these changes influence other ICU procedures and working practices [58].

Limitations of this study include the potential for poor recall from patient or relative/carer participants as the time from ICU admission to interview was not recorded[52 59]. However, as participants were usually recruited at their first ICU follow up appointment, this was unlikely to be an extended time. Furthermore, language limitations of some participants sometimes made it difficult to discern the exact point they were making during analysis, therefore although this demonstrates diversity within the sample, some finer detail may have been lost. The method of approach may have meant that more patients actively engaged in the issues being evaluated were recruited. Those patients not attending follow up appointments may have had different opinions. Pragmatic restrictions meant few family members were recruited and more patients who had experienced one of the ICU sites were involved. Finally, the application of these findings to other areas should consider that participants were included from sites in one city.

In conclusion, this exploration of a range of clinician and patient perspective suggested a patient-centred, interdisciplinary approach to implementing ICU physical rehabilitation. These findings constitute a starting point for optimising rehabilitation delivery through improvement and implementation science.

Acknowledgements

We thank staff at the research site for their support in facilitating recruitment and data collection. Data from this study has previously been presented at the Intensive Care Society State of the Art meeting 2018 and 2021, the 6th European Conference on Weaning and

For peer review only

Rehabilitation in Critically Ill Patients 2018, Physiotherapy UK conference 2018 and the North West London Research Symposium for Health Professions 2018.

Funding statement

This report is independent research arising from a Clinical Doctoral Research Fellowship, awarded to HRW, (ICA-CDRF-2015-01-026), supported by the National Institute for Health Research (NIHR) and Health Education England. The views expressed in this publication are those of the authors and not necessarily those of the NHS, the NIHR, Health Education England or the Department of Health. We acknowledge the support of the NIHR Clinical Research Network and infrastructure support for this research was provided by the NIHR Imperial Biomedical Research Centre (BRC). HRW is supported by the NIHR Imperial BRC.

Competing interests statement

HRW reports personal lecture fees from Brunel University and the University of East London. CN reports personal lecture fees from Janssen Pharmaceuticals and Web MD. CMA is a National Institute of Health Research PhD fellowship committee member, although not at the time that funding was awarded for this study. ACG reports salary support from an NIHR Research Professorship (RP-2015-06-018) and research support from the NIHR Imperial BRC.

Author contributions

HRW, SJB, CMA and ACG contributed to the conception and planning of this work. HRW, CN, MJ, SJB, CMA and ACG contributed substantially to the design of this work. HRW carried out recruitment of participants with assistance from SJB. HRW also completed data collection and CN, MJ, SJB, CMA and ACG advised on the conduct of the study. HRW had main responsibility for carrying out analysis; MJ, CN and CMA assisted in checking development of themes and HRW, CN, MJ, SJB, CMA and ACG advised on interpretation of data. HRW drafted this report, and all authors revised it critically for important content and approved the final published version. All authors are accountable for the accuracy and integrity of the work.

Ethics approval

For peer review only

This study involves human participants and was approved by the London – Bloomsbury Research Ethics Committee (17/LO/0362). Participants gave informed consent to participate in the study before taking part.

Data availability statement

No data are available.

References

1. Puthucheary ZA, Rawal J, McPhail M, et al. Acute skeletal muscle wasting in critical illness. *JAMA* 2013;310:1591-600.
2. Fan E, Dowdy DW, Colantuoni E, et al. Physical complications in acute lung injury survivors: a two-year longitudinal prospective study. *Crit Care Med* 2014;42:849-59.
3. Latronico N, Herridge M, Hopkins RO, et al. The ICM research agenda on intensive care unit-acquired weakness. *Intensive Care Med* 2017;43:1270-81.
4. Herridge MS, Tansey CM, Matte A, et al. Functional disability 5 years after acute respiratory distress syndrome. *N Engl J Med* 2011;364:1293-304.
5. Hodgson CL, Udy AA, Bailey M, et al. The impact of disability in survivors of critical illness. *Intensive Care Med* 2017;43:992-1001.
6. Bailey P, Thomsen GE, Spuhler VJ, et al. Early activity is feasible and safe in respiratory failure patients. *Crit Care Med* 2007;35:139-45.
7. Clarissa C, Salisbury L, Rodgers S, et al. Early mobilisation in mechanically ventilated patients: a systematic integrative review of definitions and activities. *J Intensive Care* 2019;7:3.
8. Hodgson CL, Stiller K, Needham DM, et al. Expert consensus and recommendations on safety criteria for active mobilization of mechanically ventilated critically ill adults. *Crit Care* 2014;18:658.
9. Wang YT, Lang JK, Haines KJ, et al. Physical Rehabilitation in the ICU: A Systematic Review and Meta-Analysis. *Crit Care Med* 2021. https://www.ncbi.nlm.nih.gov/pubmed/34406169 (accessed 8th January 2022).
10. Team Study Investigators, Anzics Clinical Trials Group, Hodgson CL, et al. Early Active Mobilization during Mechanical Ventilation in the ICU. *N Engl J Med* 2022;387:1747-58.
11. Connolly BA, Mortimore JL, Douiri A, et al. Low Levels of Physical Activity During Critical Illness and Weaning: The Evidence-Reality Gap. *J Intensive Care Med* 2019;34:818-27.
12. Nydahl P, Ruhl AP, Bartoszek G, et al. Early mobilization of mechanically ventilated patients: a 1-day point-prevalence study in Germany. *Crit Care Med* 2014;42:1178-86.
13. Bakhru RN, Wiebe DJ, McWilliams DJ, et al. An Environmental Scan for Early Mobilization Practices in U.S. ICUs. *Crit Care Med* 2015;43:2360-9.
14. Berney SC, Harrold M, Webb SA, et al. Intensive care unit mobility practices in Australia and New Zealand: a point prevalence study. *Crit Care Resusc* 2013;15:260-5.
15. Jolley SE, Moss M, Needham DM, et al. Point Prevalence Study of Mobilization Practices for Acute Respiratory Failure Patients in the United States. *Crit Care Med* 2017;45:205-15.
16. Sibilla A, Nydahl P, Greco N, et al. Mobilization of Mechanically Ventilated Patients in Switzerland. *J Intensive Care Med* 2020;35:55-62.
17. Timenetsky KT, Neto AS, Assunção MSC, et al. Mobilization practices in the ICU: A nationwide 1-day point- prevalence study in Brazil. *PLOS ONE* 2020;15:e0230971.
18. Parry SM, Knight LD, Connolly B, et al. Factors influencing physical activity and rehabilitation in survivors of critical illness: a systematic review of quantitative and qualitative studies. *Intensive Care Med* 2017;43:531-42.

For peer review only

19. Ormston R, Spencer L, Barnard M, et al. The foundations of qualitative research. In: Ritchie J, Lewis J, McNaughton Nicholls C, et al., eds. Qualitative research practice A guide for social science students and researchers. 2nd ed. London: SAGE 2014:1-25.
20. Barber EA, Everard T, Holland AE, et al. Barriers and facilitators to early mobilisation in Intensive Care: a qualitative study. *Aust Crit Care* 2015;28:177-82; quiz 83.
21. Anekwe DE, Milner SC, Bussieres A, et al. Intensive care unit clinicians identify many barriers to, and facilitators of, early mobilisation: a qualitative study using the Theoretical Domains Framework. *J Physiother* 2020;66:120-27.
22. Chaplin T, McLuskey J. What influences nurses' decision to mobilise the critically ill patient? *Nurs Crit Care* 2020;25:353-59.
23. Parry SM, Remedios L, Denehy L, et al. What factors affect implementation of early rehabilitation into intensive care unit practice? A qualitative study with clinicians. *J Crit Care* 2017;38:137-43.
24. Holdsworth C, Haines KJ, Francis JJ, et al. Mobilization of ventilated patients in the intensive care unit: An elicitation study using the theory of planned behavior. *J Crit Care* 2015;30:1243-50.
25. Goddard SL, Lorencatto F, Koo E, et al. Barriers and facilitators to early rehabilitation in mechanically ventilated patients-a theory-driven interview study. *J Intensive Care* 2018;6:4.
26. Krupp AE, Ehlenbach WJ, King B. Factors Nurses in the Intensive Care Unit Consider When Making Decisions About Patient Mobility. *Am J Crit Care* 2019;28:281-89.
27. Eakin MN, Ugbah L, Arnautovic T, et al. Implementing and sustaining an early rehabilitation program in a medical intensive care unit: A qualitative analysis. *J Crit Care* 2015;30:698-704.
28. Curtis L, Irwin J. Ambulation of patients who are mechanically ventilated: nurses' views. *Nurs Manag* 2017;24:34-39.
29. Williams N, Flynn M. An exploratory study of physiotherapists' views of early rehabilitation in critically ill patients. *Physiother Pract Res* 2013;34:93-102.
30. van Willigen Z, Ostler C, Thackray D, et al. Patient and family experience of physical rehabilitation on the intensive care unit: a qualitative exploration. *Physiotherapy* 2020;109:102-10.
31. Ritchie J, Lewis J, McNaughton Nicholls C, et al. Qualitative research practice. A guide for social science students and researchers. 2nd ed. London: SAGE 2014.
32. Tong A, Sainsbury P, Craig J. Consolidated criteria for reporting qualitative research (COREQ): a 32-item checklist for interviews and focus groups. *Int J Qual Health Care* 2007;19:349-57.
33. Bryman A. Social research methods. 5th ed. Oxford: Oxford University Press 2016.
34. Ritchie J, Lewis J, Elam G, et al. Designing and selecting samples. In: Ritchie J, Lewis J, McNaughton Nicholls C, et al., eds. Qualitative research practice A guide for social science students and researchers. 2nd ed. London: SAGE 2014:111-45.
35. Britten N. Qualitative interviews in medical research. *BMJ* 1995;311:251-3.
36. Doody O, Noonan M. Preparing and conducting interviews to collect data. *Nurse Res* 2013;20:28-32.
37. Yeo A, Legard R, Keegan J, et al. In-depth interviews. In: Ritchie J, Lewis J, McNaughton Nicholls C, et al., eds. Qualitative research practice A guide for social science students and researchers. 2nd ed. London: SAGE 2014.
38. Strauss A, Corbin J. Basics of qualitative research. Techniques and procedures for developing grounded theory. 2nd ed. London: SAGE Publications 1998.
39. Dubb R, Nydahl P, Hermes C, et al. Barriers and Strategies for Early Mobilization of Patients in Intensive Care Units. *Ann Am Thorac Soc* 2016;13:724-30.
40. McConnell-Henry T, Chapman Y, Francis K. Member checking and Heideggerian phenomenology: a redundant component. *Nurse Res* 2011;18:28-37.
41. Spencer L, Ritchie J, Ormston R, et al. Analysis: Principles and processes. In: Ritchie J, Lewis J, McNaughton Nicholls C, et al., eds. Qualitative research practice A guide for social science students and researchers. 2nd ed. London: SAGE 2014:269-93.

For peer review only

42. Spencer L, Ritchie J, O'Connor W, et al. Analysis in practice. In: Ritchie J, Lewis J, McNaughton Nicholls C, et al., eds. Qualitative research practice A guide for social science students and researchers. 2nd ed. London: SAGE 2014:295-345.
43. Choi BC, Pak AW. Multidisciplinarity, interdisciplinarity and transdisciplinarity in health research, services, education and policy: 1. Definitions, objectives, and evidence of effectiveness. *Clin Invest Med* 2006;29:351-64.
44. Korner M. Interprofessional teamwork in medical rehabilitation: a comparison of multidisciplinary and interdisciplinary team approach. *Clin Rehabil* 2010;24:745-55.
45. Jennings C, Astin F. A multidisciplinary approach to prevention. *Eur J Prev Cardiol* 2017;24:77-87.
46. Bronstein LR. A model for interdisciplinary collaboration. *Soc Work* 2003;48:297-306.
47. Petri L. Concept analysis of interdisciplinary collaboration. *Nurs Forum* 2010;45:73-82.
48. Irwin RS, Flaherty HM, French CT, et al. Interdisciplinary collaboration: the slogan that must be achieved for models of delivering critical care to be successful. *Chest* 2012;142:1611-19.
49. Urisman T, Garcia A, Harris HW. Impact of surgical intensive care unit interdisciplinary rounds on interprofessional collaboration and quality of care: Mixed qualitative-quantitative study. *Intensive Crit Care Nurs* 2018;44:18-23.
50. Liew SM, Mordiffi SZ, Ong YJA, et al. Nurses' perceptions of early mobilisation in the adult Intensive Care Unit: A qualitative study. *Intensive Crit Care Nurs* 2021;66:103039.
51. Soderberg A, Karlsson V, Ahlberg BM, et al. From fear to fight: Patients experiences of early mobilization in intensive care. A qualitative interview study. *Physiother Theory Pract* 2020. https://www.tandfonline.com/doi/full/10.1080/09593985.2020.1799460 (accessed 12 July 2021).
52. Corner EJ, Murray EJ, Brett SJ. Qualitative, grounded theory exploration of patients' experience of early mobilisation, rehabilitation and recovery after critical illness. *BMJ Open* 2019;9:e026348.
53. D'Lima DM, Murray EJ, Brett SJ. Perceptions of Risk and Safety in the ICU: A Qualitative Study of Cognitive Processes Relating to Staffing. *Crit Care Med* 2018;46:60-70.
54. Laerkner E, Egerod I, Olesen F, et al. Negotiated mobilisation: An ethnographic exploration of nurse-patient interactions in an intensive care unit. *J Clin Nurs* 2019;28:2329-39.
55. Heyland DK, Davidson J, Skrobik Y, et al. Improving partnerships with family members of ICU patients: study protocol for a randomized controlled trial. *Trials* 2018;19:3.
56. Marshall AP, Wake E, Weisbrodt L, et al. A multi-faceted, family-centred nutrition intervention to optimise nutrition intake of critically ill patients: The OPTICS feasibility study. *Aust Crit Care* 2016;29:68-76.
57. Haines KJ. Engaging Families in Rehabilitation of People Who Are Critically Ill: An Underutilized Resource. *Phys Ther* 2018;98:737-44.
58. Skivington K, Matthews L, Simpson SA, et al. A new framework for developing and evaluating complex interventions: update of Medical Research Council guidance. *BMJ* 2021;374:n2061.
59. Nickels MR, Aitken LM, Barnett AG, et al. Acceptability, safety, and feasibility of in-bed cycling with critically ill patients. *Aust Crit Care* 2020;33:236-43.

For peer review only

Consolidated criteria for reporting qualitative research checklist

Adapted from: TONG, A., SAINSBURY, P. & CRAIG, J. 2007. Consolidated criteria for reporting qualitative research (COREQ): a 32-item checklist for interviews and focus groups. Int J Qual Health Care, 19, 349-57.

Topic	Item no.	Guide questions/ descriptions	Reported on page no.
**Domain 1: Research team and reflexivity**			
Personal Characteristics			
Interviewer/facilitator	1	Which author/s conducted the interview or focus group?	6
Credentials	2	What were the researcher's credentials? E.g. PhD, MD	6
Occupation	3	What was their occupation at the time of the study?	6-7
Gender	4	Was the researcher male or female?	6
Experience and training	5	What experience or training did the researcher have?	6-7
Relationship with participants			
Relationship established	6	Was a relationship established prior to study commencement?	6-7
Participant knowledge of the interviewer	7	What did the participants know about the researcher? e.g. personal goals, reasons for doing the research	6-7
Interviewer characteristics	8	What characteristics were reported about the interviewer/facilitator? e.g. Bias, assumptions, reasons and interests in the research topic	6-7
**Domain 2: study design**			
Theoretical framework			
Methodological orientation and Theory	9	What methodological orientation was stated to underpin the study? e.g. grounded theory, discourse analysis, ethnography, phenomenology, content analysis	4
Participant selection			
Sampling	10	How were participants selected? e.g. purposive, convenience, consecutive, snowball	5
Method of approach	11	How were participants approached? e.g. face-to-face, telephone, mail, email	5
Sample size	12	How many participants were in the study?	8
Non-participation	13	How many people refused to participate or dropped out? Reasons?	8
Setting			
Setting of data collection	14	Where was the data collected? e.g. home, clinic, workplace	6
Presence of non-participants	15	Was anyone else present besides the participants and researchers?	6
Description of sample	16	What are the important characteristics of the sample? e.g. demographic data, date	9-10
Data collection			

For peer review only

Interview guide	17	Were questions, prompts, guides provided by the authors? Was it pilot tested?	6
Repeat interviews	18	Were repeat interviews carried out? If yes, how many?	6
Audio/visual recording	19	Did the research use audio or visual recording to collect the data?	6
Field notes	20	Were field notes made during and/or after the interview or focus group?	7
Duration	21	What was the duration of the interviews or focus group?	8
Data saturation	22	Was data saturation discussed?	5-6
Transcripts returned	23	Were transcripts returned to participants for comment and/or correction?	6
**Domain 3: analysis and findings**			
Data analysis			
Number of data coders	24	How many data coders coded the data?	7
Description of the coding tree	25	Did authors provide a description of the coding tree?	Supplementary material
Derivation of themes	26	Were themes identified in advance or derived from the data?	7
Software	27	What software, if applicable, was used to manage the data?	7
Participant checking	28	Did participants provide feedback on the findings?	5-6
Reporting			
Quotations presented	29	Were participant quotations presented to illustrate the themes / findings? Was each quotation identified? e.g. participant number	11-17, supplementary material
Data and findings consistent	30	Was there consistency between the data presented and the findings?	10-18, supplementary material
Clarity of major themes	31	Were major themes clearly presented in the findings?	10-18, supplementary material
Clarity of minor themes	32	Is there a description of diverse cases or discussion of minor themes?	10-18

For peer review only

Supplemental file 2

1. Patient / relative / carer topic guide.

2. Initial thematic framework

3. Table with summary of themes, sub-categories and verbatim quotes with participant numbers

For peer review only

Interview topic guide: Patient representative

Introduction:

- We value your opinions/what you think about these questions; there's not necessarily a right or wrong answer; it's not a test.

Examples of general probes that may be used

- Tell me more about that.
- Why do you think that?
- Have you got any examples?

If unable to answer open question:

- Some people have said this … [e.g. a known barrier from the literature if not already mentioned – see below for specific examples] …what do you think?

If participant unable to define early mobilisation:

- We are defining early mobilisation as something the patient does with 'their own muscle strength and control' including activities such as:
    - Moving in bed
    - Exercises
    - Sitting on the edge of the bed,
    - Standing
    - Marching on the spot,
    - Transferring from bed to chair
    - Walking…

…all whilst patients are on intensive care.

For peer review only - http://bmjopen.bmj.com/site/about/guidelines.xhtml

Interview questions

General questions:

1. Please tell me about your experience on the ICU.

2. Please tell me about physiotherapy and mobilisation (moving around and getting up and out of bed) on the ICU.
    a. What you would think of as early mobilisation?
    b. What early mobilisation did you do? How often did you do it?
    c. How was it decided that it was the right time for you to start mobilising on the ICU?

3. Do you think that early mobilisation was carried out enough?

Focused questions:

4. When you were asked if you wanted to mobilise, did you have any concerns over risks or problems that might occur?
   Potential question-specific prompts: not being well enough; fall; lines and drains/breathing tube falling out.

5. Was there anything that stopped or delayed you from mobilising on the ICU?

   Potential question-specific prompts: Feeling too unwell; lines and tubes; not enough staff.

6. What kind of things stopped mobilisation from happening more often on the ICU you were on?

For peer review only

Potential question-specific prompts: Not a priority; team did not work together; team did not have enough teaching.

7. Why do you think the staff on you ICU wanted you to mobilise and what do you think the benefits were?

   Example of potential question-specific prompts: Physical benefits; leave ICU sooner, prevent long-term complications.

8. In your experience, what things helped you to mobilise on the ICU?

   Potential question-specific prompts: Different professions working together; feeling well.

9. What kind of things do you think could be improved or changed to help mobilisation to happen more often on your ICU and to overcome some of the problems you mentioned earlier?

   Potential question-specific prompts: Better team communication; staff getting more teaching.

10. This question will explore other areas that previous interviews have brought up as important: e.g. other people have mentioned this… what do you think?

11. Is there anything else you'd like to say about what stops early mobilisation on the ICU and what could make it happen more often?

For peer review only

Initial thematic framework.

BARRIERS

1. Perceived risk of mobilising certain patients [SAFETY/RISK]

1.1 Airway and attachments

1.2 Patient instability

1.3 Patient type

1.4 Patient cognitive state

1.5 Patient medical status

1.6 Clinicians' perception of readiness to mobilise

1.7 Other

2 Patient's or their family member's reluctance for mobilisation

2.1 Clinician opinion of patient's or their family member's reluctance for mobilisation

2.2 Patients not feeling ready or motivated for mobilisation

2.3 Poor communication from clinicians

2.4 Aspects of the ICU environment not promoting mobilisation to patients

2.5 Other

3 Team working and unit culture/staff experience/ resources

3.1 Culture/Lesser priority

3.2 Roles and responsibilities

3.3 Lack of leadership

3.4 Staff experience

3.5 Lack of knowledge

3.6 Lack of resources

3.7 Logistics/ Other interventions

3.8 Nurse environment e.g. HDU/toilet

3.9 Other

FACILITATORS

4 Practical changes to how mobilisation was carried out.

4.1 Patient and family engagement

4.2 Mob treatment specific/functional rehabilitation

4.3 Use of protocols to facilitate clinical implementation of mobilisation

4.4 Patient opinion on how clinicians should communicate with them.

4.5 Specific patient motivators

For peer review only

4.6 Equipment/environment

4.7 Other changes/optimal practice

5. Improvements in team working and culture/clinician specific

5.1 Leadership

5.2 Team Communication

5.3 Experienced staff

5.4 Improved staffing/resources

5.5 Other team working

5.6 Prioritise

5.7 Education

5.8 Other culture change

6. Patient characteristics that made it easier to mobilise

6.1 Pre-morbid/general characteristics status

6.2 Acute/admission-related status

6.3 Other

7. Risks, benefits and other

7.1 Risks

7.2 Benefits

7.3 Other/irrelevant

For peer review only

Table: *Summary of themes and sub-categories*

Themes	Sub-categories	Quote number	Participant quote
1. Safety and physiological concerns	1.1 Airway, lines and attachments	1	*"I can't think who, they said an intensive care patient looks like little spiders in a web, and I agree with it. Like literally they have got tubes and attachments out everywhere." (Therapist 1)*
	1.2 Particular patient groups	2	*"…the types of patients we have have multiple and complex injuries, they're not straightforward patients to mobilise anyway…" (Therapist 3)*
	1.3 Physiological instability or dependence on organ support	3	*"…it's mainly blood pressure related for me, or their resp[iratory] rate. If I don't think they're going to tolerate mobilising, and if it's going to cause more harm than good." (Therapist 2)*
		4	*"So anybody who's on an inotrope vasopressor is, as far as I'm concerned, not safe to be mobilised… they're more likely to have a postural hypertension that would result in injury to them." (Doctor 2)*
		5	*"…whilst in itself [vasoactive drugs are] often not a reason to prevent ongoing rehab especially in junior staff it's a significant source of anxiety of doubling or trebling the dose of a medicine to keep your blood pressure up, without some form of kind of very clear guidance and encouragement that this is okay and it will return to normal following [rehabilitation] treatment." (Nurse 5)*
	1.4 Patient's ability to actively participate	6	*"And after that incident I think that was the first time I actually cried, because it hit me that "Yes, the nurses are right, I am not able to just get up and move like I would if I had been healthy," you know, so that was very traumatic for me…" (Patient 11).*
		7	*"I think I would have felt very vulnerable anyhow, [be]cause suddenly you are just weak as a baby." (Patient 7)*
		8	*"Their cognitive state is a massive thing as well. How alert are they if they've only just been woken up from sedation or if they've had a neurological event or, you know, whatever reason, that could affect their cognition." (Therapist 6)*
	1.5 Clinician perception of readiness to begin rehabilitation	9	*"…the perception, [clinicians] might think that, because this patient is dependent on a particular type of organ support, this patient is not suitable for mobilisation. So these boundaries and barriers needs to be broken." (Doctor 3)*
		10	*"I think it's probably the fear of the unwell patient, you know, we, they're in ITU therefore they must be the most unwell people in the hospital. And I think it's that kind of mentality and the fact we attach them to fifteen hundred things…" (Therapist 7)*

For peer review only

		11	*"…happy to cause no harm, or kind of, and no perceived harm by not mobilising someone but actively getting up and causing harm is a, always going to be a significant anxiety for staff…" (Nurse 5)*
		12	*"We love keeping the numbers normal, we love the sense of security that we maintain as normal physiology as we can, so that is why, a junior nurse would be more worried if she gets any change in the patient's state after mobilisation…" (Doctor 1)*
		13	*"…intensive care doctors and nurses may also be quite, well, I shouldn't say "quite" but there is a part in us that is controlling the situation and so, you know, trying to mobilise the patient may also be a bit of a paradigm shift in our own mind of, you know, this complete control over the situation and over this patient." (Doctor 4)*
		14	*"…so we kind of reset our expectations about normality and that is doing some sort of exercise when they ambulate because it is – it is to a critically ill patient it is an exercise, that we may see some events happen and as long as it is the range of acceptance, we can just modify our targets, and continue to mobilise." (Doctor 1)*
2. Patient participation and engagement	2.1 Patient reluctance to participate in rehabilitation	15	*"...well I'm in ICU... you're having intensive care, don't rock the boat by making things worse by trying to get out of bed." (Patient 8)*
		16	*"…there were times when I simply didn't want to do it… Depression, … lack of energy, lack of spirits really …" (Patient 7)*
	2.2 Communication between patients and clinicians	17	*"I would say to any nurse or any staff working in ICU… keep up that reassurance with patients because it's quite a scary experience…" (Patient 8)*
		18	*"[The consultant] pushed me beyond what I mentally thought was physically possible. I didn't believe that I could do that and of course, perhaps it's the nature of my personality, but I responded to that. Others may not have responded to that, I can't say." (Patient 3)*
	2.3 Patient engagement in planning rehabilitation	19	*"So, alongside that, we've also made like goal setting sheets that can go up by the patient's bed, so then when they sit up, when they sit upright in bed, they can see them. I draw a smiley face when they've completed one…" (Therapist 2)*
	2.4 Including activities meaningful to patients	20	*"Looking at therapy in a slightly different way and finding an activity that's meaningful to [patients], whether that's personal care or leisure activities, and through that encouraging them to… engage in that activity and then helping them to see the therapeutic value of that." (Therapist 4)*
	2.5 Identify key patient motivators	21	*"…if they can see what's in it for them, that they're gaining in dignity and all of that, they might cooperate more." (Relative 2)*

For peer review only

	2.6 The role of family	22	*"I remember the first time I took a few steps, the nurse said to me, "Well we'll do it with your husband," so my husband stood on one side and said, "We'll go for a walk with your husband," ... So it was most amazing feeling ever, you know? So everything, kind of in my head everything shut down; the nurse went away, the ward went away, it was just me and my husband going for a walk." (Patient 11)*
3. Clinician experience and knowledge	3.1 Amount of experience and support	23	*"I've had instances where it's mostly been junior people and it's terrifying. But for someone then to have a senior position helping you, that's so much better." (Nurse 2)*
	3.2 Lack of training, knowledge and skills	24	*"It doesn't happen because… we are not aware enough yet how important it is, or how much difference it could make, so it's not embedded in our thinking and in our behaviour well…" (Doctor 4)*
	3.3 Interdisciplinary team education and training	25	*"The education as well is important because you need to get people to understand what they're doing and to value it, so that they do it with passion and with skill." (Doctor 1)*
4. Teamwork	4.1 Team culture and attitudes	26	*"But a lot of it's just to do with the attitude of the individual staff member, how proactive they are and how much they believe in mobilisation as a kind of key thing" (Nurse 5)*
	4.2 Perception of roles and responsibilities	27<br>28<br>29<br>30	*"…I think the consultant's role is very important and it doesn't just include saying, "Mobilise the patient". It includes making sure that mobilisation happens and making sure that the team are, like every single member of the team is comfortable and understands the decision, and the risks related to it and understands that I am there to back them up if something happens." (Doctor 1)*<br>*"I've found that it's taken a long time for me to be accepted and for them to actually accept my opinion might be right…" (Therapist 2)*<br>*"I always felt like it was, it was very much seen it was the physio job to do anything related to moving the patient so even getting them out of bed." (Therapist 7)*<br>*"...good teamwork is really helpful, and actually a really good symbiotic relationship between the nursing staff and the therapy staff is really key." (Therapist 4)*
	4.3 Definition and delivery of rehabilitation	31<br>32<br>33	*"…mobilisation for me in ITU is hoisting somebody into a chair." (Nurse 4)*<br>*"...rehabilitation is not, you know, 20 minutes with the physio or the OT every day. Really good rehabilitation is a 24 hour approach, and that – part of that is positioning a patient in bed. Part of that is ensuring the patient gets the right nutrition as well as looking at the actual physical things that they're doing." (Therapist 4)*<br>*"...a different mentality within intensive care and to think, well actually, you know, we need to begin the rehab process all together from day one, and if a patient can be encouraged to do something they should be given the time and the opportunity to do that." (Therapist 4)*

For peer review only

	4.4 Staffing and logistics	34	*"…you start breaks at 9.30, 10.00. You finish the breaks about 12.00,1.00, so then it's not until the afternoon that people are free to help." (Nurse 2)*
5. Equipment and environment	5.1 Rehabilitation equipment	35	*"…equipment wise. You know, it's the age old problem isn't it, more of it, better ways to fix it, more money, so we've got the equipment, you know, got backup hoists." (Therapist 7)*
	5.2 ICU environment	36<br><br><br>37	*"there's just something about the environment which makes you think that you need to stay in the bed and that you shouldn't be moving around… whereas on a general ward, you don't want to be in the bed, you want to get out." (Patient 8)*<br>*"…you can see some bright lights and monitors, you can hear monitors going off, but you don't have the, "Crash, bang, wallops!" that you get in a general ward... but it's a capsule and a bubble, it's a weird feeling... "People think it's like being in a spaceship" and I thought, "That's such a good description" and that's how it did feel." (Patient 8)*
6. Risks and benefits of rehabilitation on intensive care	6.1 Clinician perception of risks	38<br><br>39	*"Falls, removal of lines and tubes and then causing bleeding, vasovagal episodes, it's actually a risky thing to mobilise an ITU patient, anything can go wrong…" (Nurse 3)*<br>*"I like mobilising patients. The more attachments the better… Because I like the challenge!" (Therapist 2)*
	6.2 Patient perception of risks	40	*"I never felt scared, I felt that the physiotherapist that was orchestrating the movement was sort of holding on to me to begin with and I never felt I was going to fall down…" (Patient 1)*
	6.3 Physical benefits	41	*"It might help their movement and I feel the more they mobilise the more their muscles are good. The more you make them sit out of the bed and stand they can stand on their feet better." (Nurse 1)*
	6.4 Psychological benefits	42	*"…the important thing is you sense that you're not just lying there waiting to die. ...so you are… you are… coming back to being a human being that wants to live." (Patient 7)*

For peer review only
